# Dysregulation of the mTOR-FMRP pathway and synaptic plasticity in an environmental model of ASD

**DOI:** 10.1038/s41380-024-02805-0

**Published:** 2024-11-27

**Authors:** Muna L. Hilal, Eleonora Rosina, Giorgia Pedini, Leonardo Restivo, Claudia Bagni

**Affiliations:** 1 Department of Fundamental Neurosciences, University of Lausanne, 1005 Lausanne, Switzerland; 2 Department of Biomedicine and Prevention, University of Rome Tor Vergata, 00133 Rome, Italy; 3 Present Address: Hôpitaux du Léman, 74200 Thonon-les-Bains, France

**Keywords:** Neuroscience, Autism spectrum disorders

## Abstract

Autism Spectrum Disorder (ASD) is caused by genetic, epigenetic, and environmental factors. Mutations in the human *FMR1* gene, encoding the Fragile X Messenger Ribonucleoprotein 1 (FMRP), cause the most common monogenic form of ASD, the Fragile X Syndrome (FXS). This study explored the interaction between the *FMR1* gene and a viral-like infection as an environmental insult, focusing on the impact on core autistic-like behaviors and the mGluR1/5-mTOR pathway. Pregnant heterozygous *Fmr1* mouse females were exposed to maternal immune activation (MIA), by injecting the immunostimulant Poly (I:C) at the embryonic stage 12.5, simulating viral infections. Subsequently, ASD-like behaviors were analyzed in the adult offspring, at 8–10 weeks of age. MIA exposure in wild-type mice led to ASD-like behaviors in the adult offspring. These effects were specifically confined to the intrauterine infection, as immune activation at later stages, namely puberty (Pubertal Immune Activation, PIA) at post-natal day 35 or adulthood (Adult Immune Activation, AIA) at post-natal day 56, did not alter adult behavior. Importantly, combining the *Fmr1* mutation with MIA exposure did not intensify core autistic-like behaviors, suggesting an occlusion effect. Mechanistically, MIA provided a strong activation of the mGluR1/5-mTOR pathway, leading to increased LTP and downregulation of FMRP specifically in the hippocampus. Finally, FMRP modulates mTOR activity via TSC2. These findings further strengthen the key role of the mGluR1/5-mTOR pathway in causing ASD-like core symptoms.

## Introduction

Autism spectrum disorder (ASD) is a complex neurodevelopmental disorder characterized by a wide range of core behavioral impairments, including difficulties in social communication and interaction alongside restricted interests and repetitive behaviors. Individuals with ASD may also experience comorbid symptoms, such as attention-deficit hyperactivity disorder (ADHD) and anxiety [1]. Worldwide, approximately 1/100 children are diagnosed with ASD [2]. ASD arises from a complex interplay between genetic and environmental factors. The genetic component contributing to ASD is estimated to be over 70% [3, 4]. Different environmental factors occurring during pregnancy, such as infection, toxic exposure or stress, contribute to the manifestation of ASD-like phenotypes in genetically predisposed individuals [5, 6]. Compared to genetic studies, environmental risk factors for ASD are not well understood, due to methodological limitations in monitoring all variables in humans [7]. Preclinical and clinical studies showed that prenatal environmental insults, such as maternal infections, increase the risk of ASD in the offspring and contribute to the onset of neurodevelopmental disorders (NDDs) exacerbating their severity [8–10]. Maternal Immune Activation (MIA) has gained attention as an environmental contributor to ASD. Although human data linking MIA to NDDs are still limited [11], studies on in-vitro and in-vivo preclinical models of prenatal immune challenge, generated by stimulating the maternal immune system with viral or bacterial agents, or inflammatory cytokines, support the “MIA hypothesis of ASD” [12–16]. Specifically, injections during gestation with polyinosinic-polycytidylic acid (Poly I:C) or lipopolysaccharides (LPS), to mimic viral or bacterial infections, respectively, lead to core ASD-like symptoms in the adult offspring, as well as brain anatomical changes, impaired brain vessel formation, structural and functional neuronal alterations including mitochondrial dysfunctions [15–19].

The most widespread monogenic cause of Autism, accounting for 1 to 6% of all ASD cases, is the Fragile X Syndrome (FXS) [20, 21]. FXS is caused by mutations in the *FMR1* gene, encoding the Fragile X Messenger Ribonucleoprotein 1 (FMRP). Individuals with FXS display a broad range of symptoms, including intellectual disabilities, anxiety, and ADHD. Approximately 60% of male individuals with FXS exhibit autistic features and behaviors [22] as well as altered brain development across different brain regions [23, 24]. FMRP is an RNA-Binding Protein highly expressed in the brain, playing a prominent role in the regulation of mRNA metabolism critical for brain development [25]. Of note, mitochondrial functions are also impaired in FXS [26–28]. A significant number of FMRP mRNA targets overlap with ASD candidate genes, suggesting a connection between FMRP and ASD [29, 30], reviewed in [25, 31, 32]. Altered FMRP levels in individuals with idiopathic and syndromic ASD indicate shared dysregulated molecular pathways between FXS and ASD [33].

Despite being a genetically well-characterized syndrome, individuals with FXS show a considerable variety of clinical symptoms. Because the heterogeneous methylation status of the *FMR1* gene alone cannot fully explain the diverse manifestations of FXS, other factors such as stressors and inflammation during pregnancy, could contribute to the observed clinical heterogeneity. Notably, the transcriptome profile of fetal brains exposed to MIA reveals a downregulation of a subset of FMRP target mRNAs [34]. In addition, prenatal exposure to bacterial LPS leads to perturbed metabotropic glutamate receptor-mediated long-term depression (mGluR-LTD) in the rat hippocampus, similarly to what has been observed in FXS [25, 35].

Despite the evidence of shared comorbidity between FXS and ASD, whether and how an immunological insult, such as MIA and post-natal immune activation (PIA), can interact with the FXS genetic background, thereby leading to a variety of clinical presentations, has not been addressed yet. Additionally, to identify vulnerable developmental windows to insults, we examined the effects of immune activation in wild-type (WT) mice during adulthood (AIA).

Here, we demonstrate that the absence of FMRP and MIA exposure both converge to core autistic-like symptoms in adult offspring, with no synergistic effect of MIA exposure on *Fmr1* knockout (KO) mice, suggesting an occlusion effect. In WT mice, only MIA exposure promotes the emergence of ASD-like behaviors, as exposure to immune activation during puberty (PIA) or adulthood (AIA) does not cause behavioral deficits. WT adult offspring exposed to MIA show a significant decrease in FMRP levels specifically in the hippocampus, while PIA does not affect its expression. Both MIA and *Fmr1* mutation independently disrupt the hippocampal mGluR1/5-dependent LTD and the mTOR signaling pathway in the adult offspring. Furthermore, FMRP downregulation driven by MIA leads to mTOR hyperactivation via TSC2. Altogether, these results suggest that MIA triggers a dysregulation of the mGluR1/5-mTOR-FMRP pathway, promoting the manifestation of core autistic-like traits.

## Materials and methods

### Animals

See Supplementary Information for details.

Male *Fmr1* KO mice and WT littermates on a C57BL/6J background (The Jackson Laboratory, Bar Harbor, Maine, USA) were used for all the experiments.

#### Ethical statement

All experiments were performed in accordance with institutional and national guidelines on the use of laboratory animals, approved by the Veterinary Authorities (Canton Vaud, Switzerland and Rome, Italy) and carried out in accordance with the European Communities Council Directive of 24 November 1986 (86/609EEC) under the approved animal licenses (Canton Vaud Veterinary Authorities, Switzerland: VD3150 and Istituto Superiore di Sanita’ Rome, Italy: 301/2024-PR).

### Poly (I:C) injection

See Supplementary Information for details.

*Fmr1* *heterozygous* females 8–12 weeks old were mated overnight with WT males of the same age. Poly (I:C) (Sigma-Aldrich) was prepared on the day of the injection in sterile NaCl 0.9% at a final concentration of 40 mg/ml. In total, 37 dams were randomly assigned to either 20 mg/kg Poly (I:C) or vehicle and received an intraperitoneal (*i.p.)* injection at the embryonic stage 12.5 (E12.5). Injected dams were weighed again to monitor for pregnancy loss on E13.5 and then left undisturbed until delivery of the pups. Out of 26 Poly (I:C) injected dams, 11 had miscarriages the day after the injection, such a frequency agrees with previous studies [36], resulting in no surviving pups and the loss of the entire offspring. Male offspring and littermates analyzed in this study were derived from 15 Poly (I:C) and 11 vehicle-treated dams.

### Behavioral assays

See Supplementary Information for details.

#### Ultrasonic vocalizations (USVs)

Ultrasonic vocalizations (USVs) were recorded in offspring littermates of vehicle- or MIA-treated mothers at postnatal day 4 (P4) using the Avisoft recording software (SASLab Pro system).

#### Pup retrieval test

Single pups were removed from the home cage with the mother to register ultrasonic vocalizations (USVs) in the sound-proof chamber. The retrieval test was conducted upon the reintroduction of the tested pup in the home cage with all dams. Latency required for the mother to recover the pup and bring it back to the nest was measured during the separation of mothers from the offspring.

#### Open field test & novel object exploration

The test was performed as previously described in [37] using a video tracking system (Ethovision 11.0 XT, Noldus).

#### Elevated plus maze test

The test was performed as previously described in [38]. Mice were tracked (Ethovision 11.0 XT, Noldus) to measure the time spent in the open and closed arms.

#### Marble burying test

Standard polycarbonate rat cages fitted with filter-top covers were used for the marble burying test as in [39].

#### Three chamber test

The equipment used for social preference and social novelty consists of a rectangular three-chamber box (Noldus). Mouse position was tracked (Ethovision 11.0 XT, Noldus) to measure the time spent in the interaction zone near the enclosure.

#### Pre-pulse inhibition (PPI)

The pre-pulse inhibition was performed as previously described [40]. Briefly, the mice were individually placed in the chamber (Med Associates, Inc.) and underwent an acclimation period followed by a three-block session.

#### Autistic score

A score that encompasses the analyzed core symptoms of ASD was obtained averaging the results of the different behavioral tests performed for each animal.

### Electrophysiology

See Supplementary Information for details.

Following decapitation, brains were removed from the skull and placed in cold artificial cerebrospinal fluid (aCSF) solution. Coronal brain slices of 350 μm were obtained using a vibratome (Leica Biosystems).

To record activity of CA3-CA1 synapses, we electrically stimulated Schaffer collateral fibers and recorded CA1 field excitatory postsynaptic potentials (fEPSPs). After a stable baseline for at least 20 min, 50 µM of (S)-3,5-Dihydroxyphenylglycine (DHPG) (Tocris Bioscience) was applied to the bath to induce mGluR5-dependent LTD. The slope of fEPSP was monitored for 60 min after induction, and averages of the last 10 min were compared between the different groups as described in the statistical analysis.

### Ubiquitination pull-down

See Supplementary Information for details.

Ubiquitination of FMRP was detected using a Signal-Seeker Ubiquitination Detection Kit (Cytoskeleton) according to the manufacturer’s instructions.

### Western blots

See Supplementary Information for details.

Different brain regions were dissected following decapitation and stored at −80 °C until use for biochemical analysis. Proteins (20 μg) were separated on a 4-15% Mini-PROTEAN™ TGX Stain-Free™ Protein Gels and blotted on a PVDF membrane (Roche-Merck). Membranes were incubated using the primary and the secondary antibodies listed in the Supplementary Information. Proteins were revealed using the Odyssey Infrared Imaging System or an enhanced chemiluminescence kit (Bio-Rad) and the imaging system LAS-4000 mini. All phosphoproteins were normalized relative to the total protein on the same blot. Protein levels were normalized using the average of Ponceau red staining or Coomassie blue staining and Vinculin signal on the membranes. Signal quantification was performed using ImageQuant TL software.

### Enzyme-Linked Immunosorbent Assay (ELISA)

See Supplementary Information for details.

After 3 h of vehicle or Poly (I:C) administration, blood was collected in heparin-coated tubes either from the submandibular vein or following rapid decapitation. Interleukin 6 (IL-6) and Interleukin 17a (IL-17a) were measured using an ELISA kit (IL-6, Enzo Life Sciences; IL-17a, Sigma-Aldrich) according to the manufacturer’s instructions.

### Quantification of Serum Cytokine Levels using a Luminex Platform

See Supplementary Information for details.

After 3 h of vehicle or Poly (I:C) administration, blood was collected in heparin-coated tubes either from the submandibular vein or following rapid decapitation. Mouse cytokine levels from blood serum were assessed at the ISO 9001:2015-certified Labospace Srl laboratory (Labospace, Milan, Italy). The MILLIPLEX® Mouse Cytokine/Chemokine Magnetic Bead Custom-made panel (Millipore Sigma) including Interleukin 6 (IL-6), Regulated upon Activation Normal T cell Expressed and Secreted (RANTES, or C–C motif Chemokine ligand 5, CCL5) and Tumor Necrosis Factor α (TNF-α) was used according to the manufacturer’s protocol. Data in the scatter plots represent six technical replicates of three independent biological samples.

### Polysome-mRNPs analysis

See Supplementary Information for details.

The polysome-mRNPs distribution of the *Fmr1* and *β−actin* mRNAs was performed as previously described [41].

### FMRP RNA immunoprecipitation (RIP)

See Supplementary Information for details.

FMRP RNA immunoprecipitation (RIP) was performed as previously described [24].

### RNA purification and RT-qPCR

See Supplementary Information for details and primers used.

Total RNA from 50 μg brain lysate was isolated using 1 ml TRIzol™ Reagent (Invitrogen) according to the manufacturer’s instructions. First-strand synthesis was generated using 1 μg of total RNA, p(dN)6 and 200 U/μl of  SuperScript™ III Reverse Transcriptase or M-MLV Reverse Transcriptase, buffer 5X M‐MLV reaction buffer, RNase OUT and dNTPs, according to the manufacturer’s instructions (Invitrogen). The mRNA expression level for each gene of interest was determined relative to a normalization factor (i.e., the average of the reference genes).

### Statistics

See Supplementary Information for detailed statistical tests used.

Statistical analyses were performed with GraphPad Prism software (Version 10.2.3 (347), San Diego, California, USA). Detailed descriptions of all statistical methods are available in the figure legends and in Supplementary Materials and Methods.

## Results

### MIA does not exacerbate behavioral deficits in *Fmr1* KO mice

**Fig. 1:**
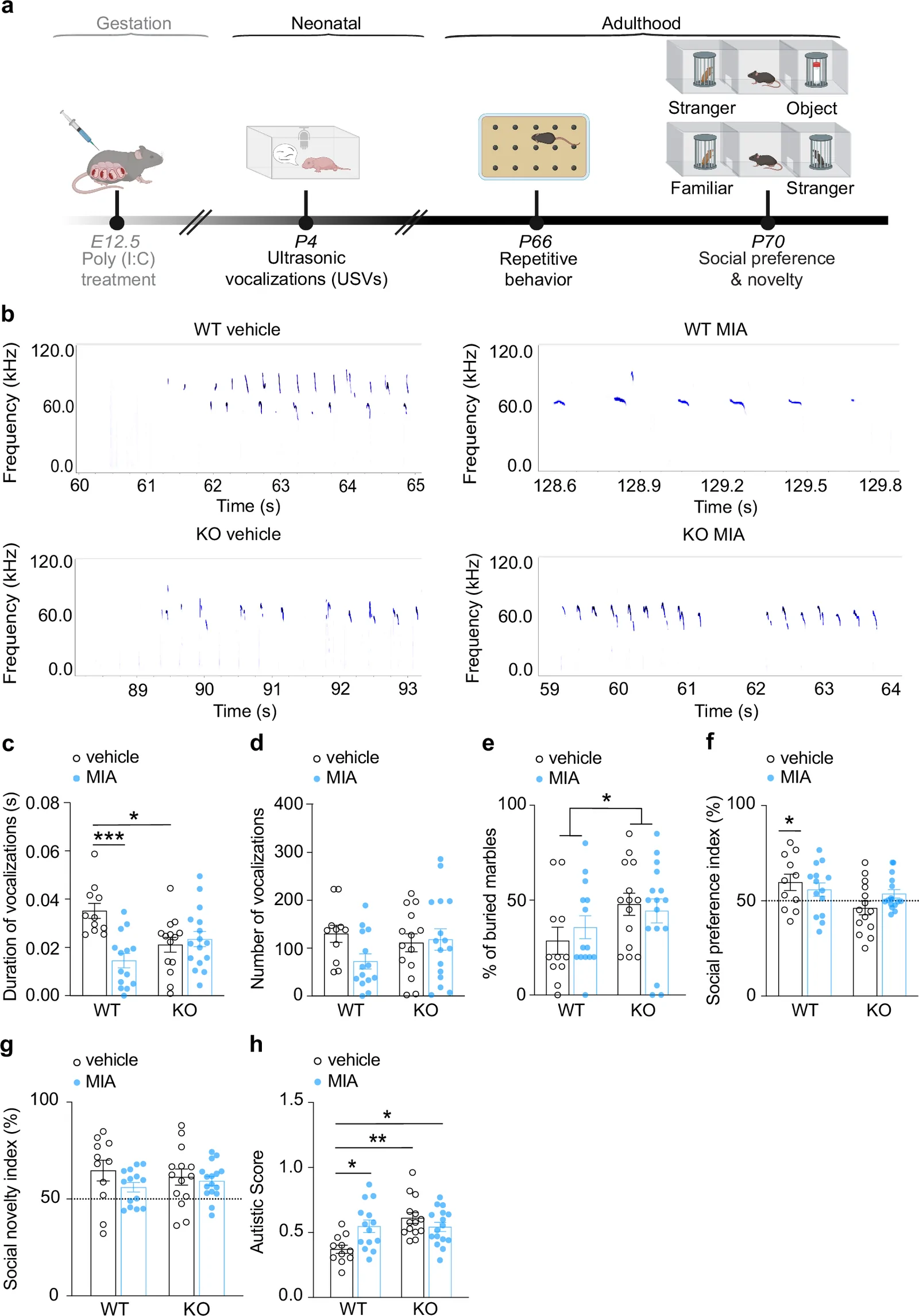
MIA does not exacerbate core autistic-like behaviors in *Fmr1* KO mice. **a** Illustration of the experiment timeline. Poly (I:C) at 20 mg/kg was administered to *Fmr1 Het* pregnant female mice at E12.5, and the offspring examined at different time points. The same cohort of animals was analyzed across the different behavioral tests. **b** Separation-induced USVs at P4. Representative pattern of USV calls for each condition. In each panel, the x axis indicates duration in seconds and the y axis shows the frequency in kHz. **c** *Fmr1* KO vehicle, *Fmr1* KO MIA and WT MIA-treated animals showed a similar decrease in vocalization compared to WT vehicle-treated group (Two-way ANOVA test, Interaction F_1.51_ = 13.39, *p* = 0.0006 and treatment effect F_1.51_ = 8.437, *p* = 0.0054, WT vehicle vs WT MIA ***p = 0.0003, WT vehicle vs KO vehicle *p = 0.02, WT vehicle vs KO MIA *p* = 0.076 in Bonferroni’s multiple comparisons test). **d** USVs at P4 showed no difference between groups in the number of emitted USVs (Two-way ANOVA test, Interaction F_1.51_ = 2.737, *p* = 0.1042 and treatment effect F_1.51_ = 1.675, *p* = 0.2014). **e** Marble burying test. Increased marble burying was observed only in the *Fmr1* KO mice, independently of the treatment (Two-way ANOVA test, Interaction F_1.51_ = 0.6801, *p* = 0.4134 and genotype effect F_1.51_ = 4.741, *p = 0.0341). **f** The three-chamber test. WT MIA-, *Fmr1* KO vehicle- and *Fmr1* KO MIA-treated mice did not exhibit any preference towards the stranger mouse (One-sample t test against chance level (50%): WT vehicle: *p = 0.0474; Three-way ANOVA test, stimulus effect F_1.51_ = 3.35, *p* = 0.073, stimulus x genotype x treatment, F_1.51_ = 0.66, *p* = 0.418). **g** Social novelty assessed with the three-chamber test showed no difference between groups (Two-way ANOVA test, Interaction F_1.51_ = 0.8640, *p* = 0.3570 and treatment effect F_1.51_ = 2.259, *p* = 0.1390). **h** Autistic-like score encompassing three core features of ASD revealed increased autistic-like traits in WT MIA-treated animals and no additional effect of the MIA treatment on *Fmr1* KO mice (Two-way ANOVA test, Interaction F_1.51_ = 9.345, *p* = 0.0036 and genotype effect F_1.51_ = 8.591, *p* = 0.0050, WT vehicle vs WT MIA *p = 0.0266, WT vehicle vs KO vehicle **p = 0.0011, WT vehicle vs KO MIA *p = 0.0268 in Bonferroni’s multiple comparisons test; WT vehicle: *n* = 11; WT MIA: *n* = 14; *Fmr1* KO vehicle: *n* = 14; *Fmr1* KO MIA: *n* = 16). Data are represented as mean ± SEM.

MIA was modeled in pregnant *Fmr1 heterozygous (Het)* female mice with a single *i.p*. injection of Poly (I:C) at E12.5. Next, ASD-like behaviors such as social interaction and USVs, in WT and *Fmr1* KO male offspring were investigated. Specifically, social communication in vehicle- or MIA-exposed offspring was tested by examining isolation-induced USVs at P4, considering that vocal signals are used as proxies to monitor communication [42] (Fig. 1a–d). USV calls in *Fmr1* KO vehicle-treated mice were significantly shorter compared to WT vehicle-treated mice (Fig. 1b, c). Similarly, the WT MIA-treated group showed a drastic reduction in the duration of vocalization compared to WT vehicle-treated mice, while MIA did not further affect USVs in *Fmr1* KO mice (Fig. 1b, c). The number of USVs was comparable in both genotypes, regardless of MIA exposure (Fig. 1d). Repetitive behaviors, evaluated in mice by testing burying behaviors [39], were increased in vehicle-treated *Fmr1* KO adult mice compared to vehicle-treated WT mice. Noteworthy, MIA exposure did not affect this repetitive behavior in either genotype (Fig. 1a, e).

Social preference and novelty were investigated in the adult offspring using the three-chamber test [43]. While the WT vehicle-treated mice displayed a significant preference towards the unfamiliar mouse over the inanimate object, the *Fmr1* KO vehicle-treated mice spent a similar amount of time exploring the object and the unfamiliar mouse (Fig. 1a, f and Supplementary Fig. S1a, b), as previously reported [44]. Interestingly, WT MIA-treated offspring did not exhibit significant social preference when compared to chance level (50%), indicating that MIA offspring explored both the object and the stranger mouse with equal interest. However, MIA did not exacerbate impaired sociability in *Fmr1* KO animals (Fig. 1a, f and Supplementary Fig. S1a, b). Finally, social novelty recognition in WT mice was not affected by MIA exposure or in the genetic model *Fmr1* KO, although WT MIA-treated animals spent comparable time with both the stranger and the familiar mouse (Fig. 1a, g and Supplementary Fig. S1c, d), consistent with previous findings in WT mice [45].

Based on the results obtained from testing the three main features of ASD (social communication, social preference, and repetitive behavior), we built a score that encompasses these core symptoms (Fig. 1h). This analysis revealed a significant increase in autistic-like core features in WT mice exposed to MIA, in *Fmr1* KO vehicle-treated and *Fmr1* KO MIA-treated mice compared to the WT vehicle-treated group (Fig. 1h). Surprisingly, *Fmr1* KO offspring of MIA-treated mothers did not exhibit an increased autistic-like score compared to the KO offspring of vehicle-treated mothers (Fig. 1h). These results suggest that the *Fmr1* mutation and MIA affect common downstream pathway(s) that cannot be further disrupted through synergistic effect when both risk factors are combined.

To assess the inflammatory response to Poly (I:C), we monitored the levels of peripheral IL-6 following Poly (I:C) injection in WT pregnant dams. Pregnancy was confirmed by a 25% weight gain at E12.5 (Supplementary Fig. S2a), indicating correct pregnancy progression at the time of the immune challenge. A dose-response curve showed increased serum IL-6 levels following a single injection of 20 mg/kg of Poly (I:C), as previously reported [46]. Lower doses (4 mg/kg) did not show differences compared to WT vehicle-treated mice. A higher dose (40 mg/kg) did not induce stronger inflammation compared to the well-established condition of 20 mg/kg of Poly (I:C) (Supplementary Fig. S2b). To further validate the MIA model using Poly (I:C), we investigated in pregnant WT and *Fmr1 Het* females the levels of pro-inflammatory cytokines/chemokines, specifically IL-17a, IL-6, RANTES (CCL5) and TNF-α, which are known to increase after Poly (I:C) administration [47–50]. MIA led to increased serum protein levels of IL-17a, IL-6, RANTES (CCL5) and TNF-α after 3 hours of Poly (I:C) injection compared to vehicle-exposed dams (Supplementary Fig. S2c–f), validating the activation of the immune system. Similar maternal immune response between WT and *Fmr1 Het* MIA-exposed females was observed, suggesting that the *Fmr1* mutation does not amplify the effect of the immune activation during pregnancy (Supplementary Fig. S2c–f). These data confirm the establishment of the immune response in pregnant animals after Poly (I:C) injection, as previously reported [47, 50]. The latency to pup retrieval in WT dams injected with either vehicle or Poly (I:C) was comparable, indicating no effect of Poly (I:C) on maternal care (Supplementary Fig. S2g), consistent with previously published work [51]. Furthermore, no differences in peripheral inflammation were observed between the adult offspring of the vehicle-treated and MIA-treated groups (Supplementary Fig. S2h) ruling out that the observed behavioral effects are solely attributable to chronic inflammation in the offspring.

**Fig. 2:**
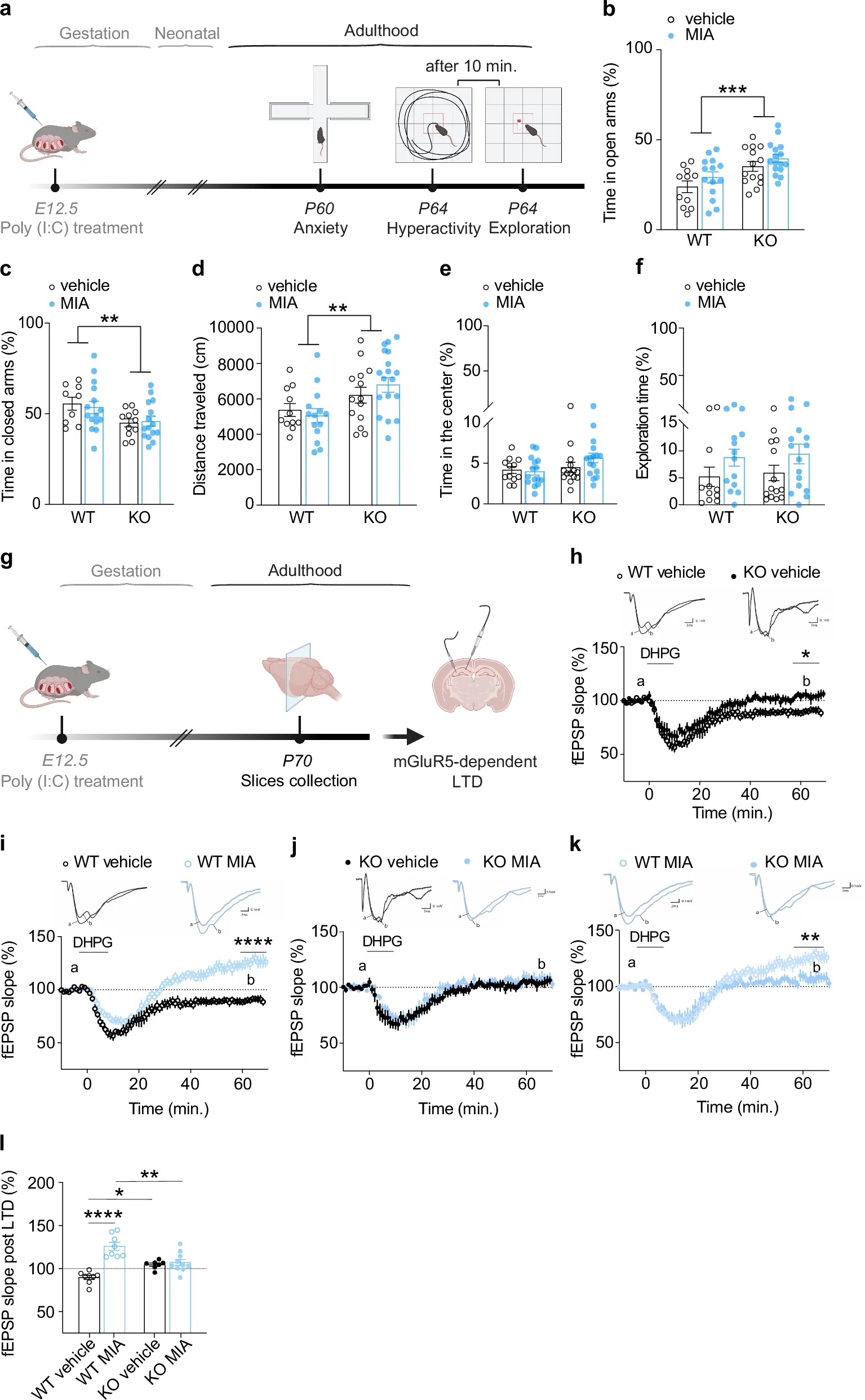
MIA treatment does not affect anxiety and hyperactivity in the *Fmr1* KO mice while disrupts mGluR1/5 signaling in both WT and *Fmr1* KO mice. **a** Illustration of the experiment timeline. Poly (I:C) at 20 mg/kg was administered to *Fmr1 Het* pregnant female mice at E12.5, and offspring was examined at different time points. The same cohort of animals, also used in Fig. 1a, was analyzed across different behavioral tests. **b** Elevated plus maze test. *Fmr1* KO mice spent more time in the open arms compared to WT mice independently of MIA exposure (Two-way ANOVA test, Interaction F_1.51_ = 0.02005, *p* = 0.8880 and genotype effect F_1.51_ = 15.51, ***p = 0.0002. **c** Elevated plus maze test. *Fmr1* KO mice spent less time in the closed arms compared to WT mice independently of MIA exposure (Two-way ANOVA test, Interaction F_1.46_ = 0.2322, *p* = 0.6322 and genotype effect F_1.46_ = 8.216, **p = 0.0062). **d** Open field test. *Fmr1* KO mice traveled longer distances compared to WT mice independently from MIA treatment (Two-way ANOVA test, Interaction F_1.51_ = 1.146, *p* = 0.2894 and genotype effect F_1.51_ = 9.386, **p = 0.0035. **e** Open field test. WT and *Fmr1* KO mice spent similar time in the center of the open field arena, without any effect of the treatment (Two-way ANOVA test, Interaction F_1.53_ = 1.515, *p* = 0.2238 and genotype effect F_1.53_ = 3.285, *p* = 0.0756). **f** The novel object exploration test was conducted in an open field arena. WT and *Fmr1* KO MIA-treated mice spent more time exploring the novel object compared to vehicle-treated mice (Two-way ANOVA test, Interaction F_1.51_ = 0.0001017, *p* = 0.9920 and treatment effect F_1.51_ = 4.461, *p = 0.0396; WT vehicle: *n* = 9–12; WT MIA: *n* = 14–15; *Fmr1* KO vehicle: *n* = 11–14; *Fmr1* KO MIA: *n* = 15–16). **g** Illustration of the experiment timeline. Poly (I:C) at 20 mg/kg was administered to *Fmr1 Het* pregnant female mice at E12.5, and offspring was examined in the adulthood. **h** Application of DHPG for 10 minutes induced mGluR1/5-dependent LTD in slices derived from WT vehicle-treated animals but not in slices from *Fmr1* KO vehicle-treated mice, as previously described (The last 10 minutes of the traces in panels **h**–**k** were compared between all conditions by Two-way ANOVA; see panel 2**l**). **i** Slices from WT MIA-treated mice displayed a significant long-term potentiation instead of LTD following application of DHPG and a significant difference between WT vehicle and WT MIA was observed. **j** Application of DHPG did not induce long-term modifications of the synaptic transmission in slices derived from *Fmr1* KO vehicle- and *Fmr1* KO MIA-treated animals. **k** Slices from WT MIA-treated and *Fmr1* KO MIA-treated animals exhibited different responses to DHPG application. “a” represents the average of 10 minutes of baseline recordings (before DHPG) while “b” is the average of the last 10 minutes recorded 1 h post application of DHPG. **l** Percentage of modification in fEPSPs following DHPG application showed significant LTD and LTP in slices from WT vehicle and WT MIA-treated mice, respectively, and no change in slices from *Fmr1* KO vehicle or *Fmr1* KO MIA (Two-way ANOVA, Interaction F_1.29_ = 23.48, *p* < 0.0001 and treatment effect F_1.29_ = 31.15, *p* < 0.0001, WT vehicle vs WT MIA ****p < 0.0001, WT vehicle vs KO vehicle *p = 0.0341, WT vehicle vs KO MIA *p* = 0.0047, WT MIA vs KO vehicle *p* = 0.0013, WT MIA vs KO MIA **p = 0.0021 in Tukey’s multiple comparisons test; WT vehicle: *n* = 8; WT MIA: *n* = 8; *Fmr1* KO vehicle: *n* = 7 – 10; *Fmr1* KO MIA: *n* = 10). Data are represented as mean ± SEM.

In humans, ASD can be associated with anxiety and hyperactivity [52]. In rodents, these traits are modeled using the elevated plus maze (EPM) and the open-field test, respectively [37, 38]. The *Fmr1* mutation increased the time spent in the open arms of the EPM compared to WT mice (Fig. 2a, b), as previously shown [53], while MIA had no significant impact on either WT or *Fmr1* KO mice (Fig. 2a, b). A genotype effect on the time spent in the closed arms was observed (Fig. 2c), consistent with the detected decrease in anxiety-like behavior in the *Fmr1* KO mice, regardless of the treatment (Fig. 2b). A similar profile was observed in the open-field test, where *Fmr1* KO mice were more hyperactive and traveled longer distances compared to WT mice, regardless of the MIA (Fig. 2a, d), as previously described in *Fmr1* KO mice [53, 54]. We did not observe any difference between genotype or treatment groups in the total time spent within the center zone compared to the outer side of the open field arena (Fig. 2a, e). We also evaluated novel object exploration in the open-field test. MIA exposure significantly increased the time spent exploring the object in both genotypes (Fig. 2a, f), consistent with previous findings in WT mice [55], while no MIA x genotype interaction effect was found.

### MIA affects the mGluR1/5 signaling causing a switch from LTD to LTP

The mGluR1/5 receptor signaling is disrupted in a mouse model of FXS [25, 56]. Similarly, MIA exposure alters mGluR-LTP in hippocampal synapses [35]. To examine whether such dysregulation is exacerbated by combining the genetic and environmental risk factors during pregnancy, we measured field excitatory postsynaptic potentials (fEPSPs) and induced mGluR1/5-dependent LTD (Fig. 2g). As expected, slices from WT vehicle-treated animals displayed LTD 60 minutes following the application of the mGluR5 agonist DHPG at the CA3-CA1 synapses (Fig. 2h, l). In slices from adult *Fmr1* KO vehicle-treated mice, DHPG had no significant long-term effect (Fig. 2h, l). While previous research in juvenile (P21-P32) *Fmr1* KO animals has shown increased LTD [57, 58], our results in adult mice (P60-P80) indicate an impaired hippocampal mGluR5-LTD, consistent with age-dependent alterations previously reported in the *Fmr1* KO mice [59, 60].

Of note, slices derived from WT mice exposed to MIA exhibited 35% induction of LTP 60 minutes after DHPG application (Fig. 2i, l). Intriguingly, the mGluR1/5-LTD in hippocampal slices from *Fmr1* KO MIA-treated animals resembled that of slices from *Fmr1* KO vehicle-treated mice (Fig. 2j, l). Moreover, an LTD-to-LTP switch was observed in slices from WT MIA-treated animals compared to WT vehicle-treated. The same electrophysiological behavior was not detected in the *Fmr1* KO MIA-treated mice (Fig. 2k, l).

### Pubertal immune activation (PIA) has a specific effect on the mGluR1/5 signaling

**Fig. 3:**
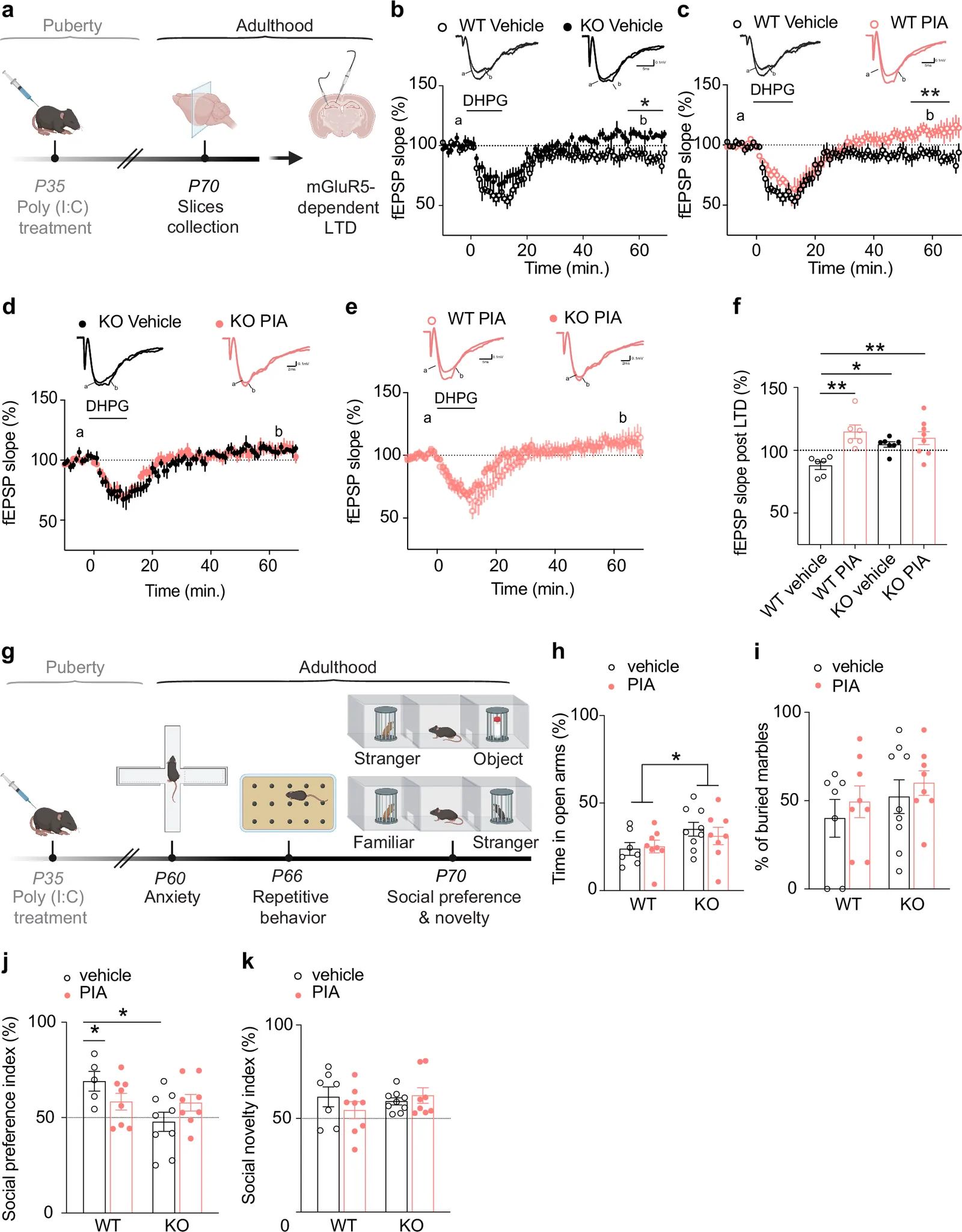
PIA disrupts mGluR1/5 signaling in WT mice and does not induce ASD-like behaviors. **a** Illustration of the experiment timeline. Poly (I:C) at 20 mg/kg was administered to WT or *Fmr1* KO male mice at P35 and outcomes were examined in the adulthood. **b** Application of DHPG for 10 minutes induced mGluR1/5-dependent LTD in slices from WT vehicle-treated animals but not in *Fmr1* KO vehicle-treated mice (The last 10 minutes of the traces in panels **b**–**e** were compared between all conditions by Two-way ANOVA; see panel 3**f**). **c** Slices from WT PIA-treated mice displayed a significant LTP following application of DHPG compared to WT vehicle. **d** Application of DHPG did not induce long-term modifications of the synaptic transmission in slices from *Fmr1* KO vehicle and PIA-treated animals. **e** Slices from WT PIA and *Fmr1* KO PIA-treated animals did not exhibit different responses to DHPG application. “a” represents the average of 10 minutes of baseline recordings (before DHPG) while “b” is the average of the last 10 minutes recorded 1 h post application of DHPG. **f** Percentage of long term modification in fEPSPs upon DHPG application showed significant LTD and LTP in slices from WT vehicle and WT PIA-treated animals, respectively, and no change in slices from *Fmr1* KO vehicle and PIA-treated mice (Two-way ANOVA, Interaction F_1.23_ = 6.397, *p* = 0.0187 and treatment effect F_1.23_ = 13.72, *p* = 0.0012, WT vehicle vs WT PIA **p = 0.0019, WT vehicle vs KO vehicle *p = 0.05, WT vehicle vs KO PIA **p = 0.0070 in Tukey’s multiple comparisons test; WT vehicle: *n* = 6; WT PIA: *n* = 6; *Fmr1* KO vehicle: *n* = 7; *Fmr1* KO PIA: *n* = 8). **g** Illustration of the experiment timeline. Poly (I:C) at 20 mg/kg was administered to WT or *Fmr1* KO male mice at P35, and behavioral tests conducted in adulthood. The same cohort of animals was analyzed across the different behavioral tests. **h** Anxiety evaluated in the Elevated Plus Maze test. *Fmr1* KO mice spent more time in the open arms compared to WT mice independently of the treatment (Two-way ANOVA, Interaction F_1.28_ = 0.4087, *p* = 0.5278 and genotype effect F_1.28_ = 4.398, *p = 0.0451). **i** Repetitive behavior investigated using the marble burying test showing no effect of PIA treatment on both genotypes (Two-way ANOVA test, Interaction F_1.28_ = 0.007589, *p* = 0.9312 and genotype effect F_1.28_ = 1.553, *p* = 0.2231). **j** Social preference in adult mice assessed in the three-chamber test revealed a reduced social preference index in *Fmr1* KO vehicle, but not in PIA-treated mice (One-sample t test against chance level (50%): WT vehicle: *p = 0.0213; Two-way ANOVA, Interaction F_1.26_ = 4.381, *p* = 0.0462 and genotype effect F_1.26_ = 4.852, *p* = 0.0367, WT vehicle vs KO vehicle *p = 0.0366 in Tukey’s multiple comparisons test). **k** Social novelty in the three-chamber test showing no difference between groups (Two-way ANOVA test, Interaction F_1.28_ = 1.648 *p* = 0.2097and genotype effect F_1.28_ = 0.4616, *p* = 0.5025; WT vehicle: *n* = 5–7; WT PIA: *n* = 8; *Fmr1* KO vehicle: *n* = 9; *Fmr1* KO PIA: *n* = 8). Data are represented as mean ± SEM.

Puberty is known to be a vulnerable period [61]. The brain continues to be fine-tuned during adolescence, and insults, stressors or immune challenges during this period might contribute to the development of mental illnesses later in life [62]. Several studies have suggested a link between schizophrenia (SCZ) and inflammation [63]. To investigate whether the susceptibility of WT mice to immune activation in inducing autistic-like behavior is extended to the pubertal period, mice received an *i.p* injection of Poly (I:C) at P35. The mGluR1/5-dependent LTD was then assessed at P70 in slices from WT and *Fmr1* KO vehicle- or PIA-treated mice (Fig. 3a). While WT vehicle-treated mice exhibited a significant LTD following the application of DHPG for 10 minutes, *Fmr1* KO vehicle-treated mice did not (Fig. 3b, f), consistent with previous findings [59]. Surprisingly, exposure to PIA completely abolished the mGluR1/5-dependent LTD in slices from WT mice, resulting in a 14% induction of LTP following DHPG application (Fig. 3c, f). PIA treatment had no effect on slices derived from *Fmr1* KO mice compared to KO vehicle-treated animals, with both groups exhibiting abolished LTD without an induction of LTP (Fig. 3d, f). Finally, there was no difference in LTP induction following DHPG between WT and KO mice exposed to PIA (Fig. 3e, f). Overall, our findings indicate that PIA does not induce a pronounced LTP increase as observed upon MIA, suggesting that hippocampal synapses are more vulnerable to immune stimulation *in utero* compared to postnatal insults.

### PIA leads to schizophrenia-like behaviors

PIA treatment induced transient body weight loss one day post-treatment in both WT and *Fmr1* KO mice, with no differences observed one week later (Supplementary Fig. S3a–c). Autistic-like behaviors were evaluated at P60 (three weeks after PIA treatment), using the same battery of behavioral tests as shown in Fig. 1a (Fig. 3g and Supplementary Fig. S3a), except for the USVs, as the biological significance of these calls would differ between early postnatal and adult stages. In the anxiety test, *Fmr1* KO mice spent more time in the open arms of the EPM and were more hyperactive in the open field test regardless of the treatment (Fig. 3g, h and Supplementary Fig. S3a, d). Repetitive behavior was not affected by the treatment, as WT and *Fmr1* KO mice buried an equal number of marbles in the marble-burying test (Fig. 3g, i). Social preference was abolished specifically in the *Fmr1* KO vehicle-treated mice, but no effect was observed following PIA treatment in both genotypes (Fig. 3g, j). All groups responded to social novelty similarly to our previous experiments (Fig. 3g, k). In addition, there was no significant difference in the object exploration time between PIA or vehicle-treated WT or *Fmr1* KO mice (Supplementary Fig. S3a, e). Finally, AIA in WT mice did not cause any significant behavioral alteration (Supplementary Fig. S4), or mGluR1/5-dependent LTD (Supplementary Fig. S4k), highlighting embryonic development as the vulnerable period.

Pubertal infections may contribute to neurodevelopmental abnormalities, including sensorimotor gating defects, a hallmark of SCZ [64], also detected in FXS as well as in MIA-exposed animals [65, 66]. In mice, sensorimotor gating is measured using the pre-pulse inhibition (PPI) paradigm [67]. *Fmr1* KO mice exhibited an overall decrease in the startle response compared to WT mice (Supplementary Fig. S3a, f). PIA impaired sensorimotor gating in WT mice but did not worsen sensorimotor gating in *Fmr1* KO animals (Supplementary Fig. S3a, g, h), as previously reported in *Fmr1* KO rats [68].

### The mTOR-FMRP pathway is dysregulated in the absence of FMRP and following exposure to MIA

**Fig. 4:**
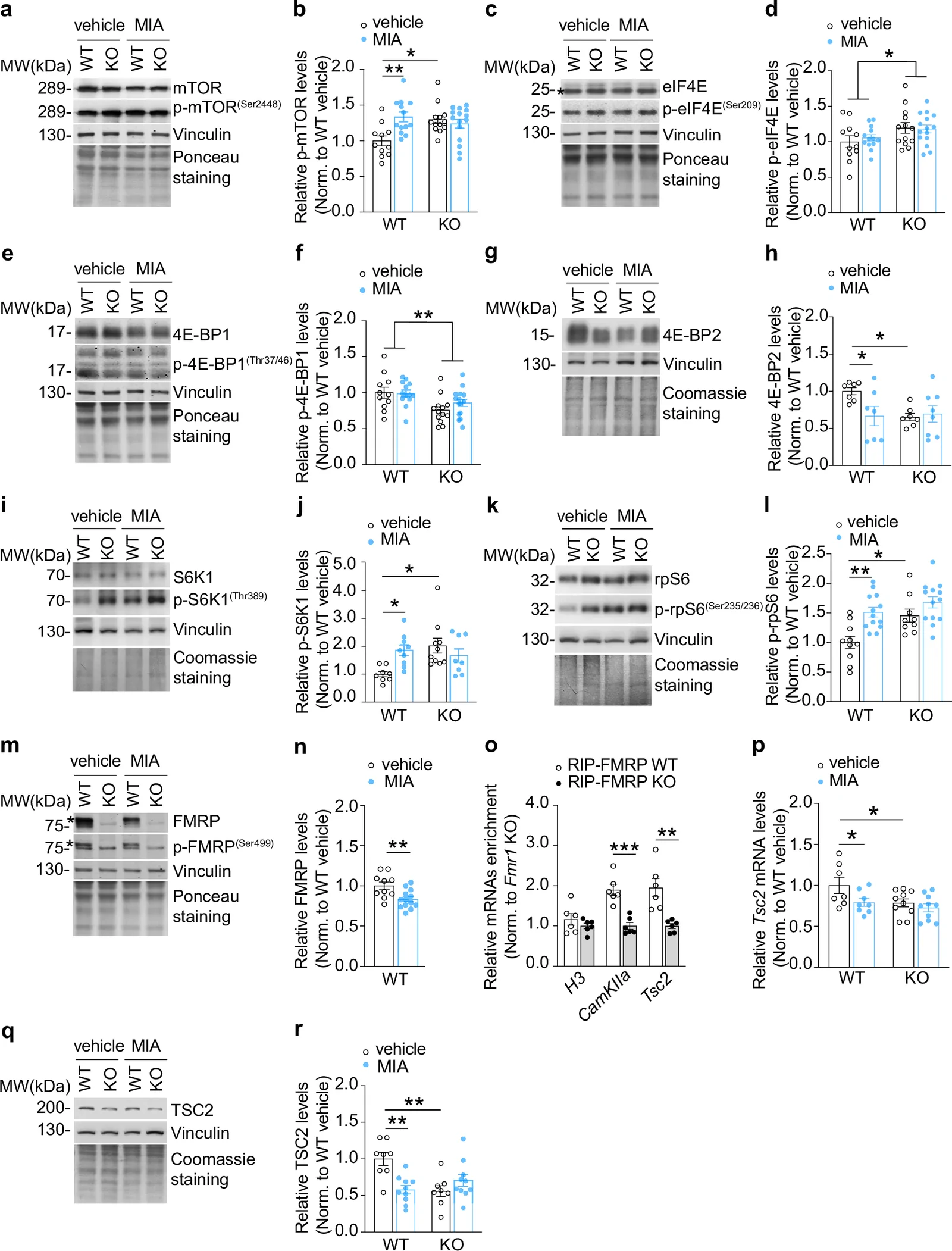
*Fmr1* mutation and exposure to MIA disrupt the mTOR-FMRP pathway. **a** Protein levels in the hippocampus of adult offspring analyzed in WT vehicle-treated, *Fmr1* KO vehicle-treated, WT MIA-treated, and *Fmr1* KO MIA-treated animals. Representative western blots for mTOR and p-mTOR (Ser2448). **b** MIA treatment and *Fmr1* mutation induced a significant increase in p-mTOR levels in the adult offspring, while MIA did not exacerbate altered mTOR activity in KO mice (Two-way ANOVA, Interaction F_1.48_ = 9.453, *p* = 0.0035 and treatment effect F_1.48_ = 4.554, *p* = 0.038, WT vehicle vs WT MIA **p = 0.0053, WT vehicle vs KO vehicle *p = 0.016 in Sidak’s multiple comparisons test). **c** Representative western blots for eIF4E and p-eIF4E (Ser209). **d** *Fmr1* mutation increased p-eIF4E levels regardless of the treatment (Two-way ANOVA, Interaction F_1.48_ = 0.2896, *p* = 0.5930 and genotype effect F_1.48_ = 6.074, *p = 0.0173). **e** Representative western blots for 4E-BP1 and p-4E-BP1 (Thr37/46). **f** p-4E-BP1 decreased in *Fmr1* KO mice, regardless of the treatment (Two-way ANOVA, Interaction F_1.48_ = 0.9875, *p* = 0.3253 and genotype effect F_1.48_ = 11.25, **p = 0.0016). **g** Representative western blots for 4E-BP2. **h** 4E-BP2 expression is reduced in *Fmr1* KO and in WT MIA-treated mice (Two-way ANOVA, Interaction F_1.25_ = 4.029, *p* = 0.0557, WT vehicle vs WT MIA *p = 0.0461, WT vehicle vs *Fmr1* KO vehicle: *p = 0.0461, in Holm-Šídák’s multiple comparisons test). **i** Representative western blots for S6K1 and p-S6K1 (Thr389). **j** S6K1 activity was enhanced in KO vehicle and WT MIA treated-mice (Two-way ANOVA, Interaction F_1.31_ = 7.626, *p* = 0.0096, WT vehicle vs WT MIA *p = 0.0491, WT vehicle vs KO vehicle *p = 0.0113 Tukey’s multiple comparisons test). **k** Representative western blots for rpS6 and p-rpS6 (Ser235/236). **l** p-rpS6 increased in WT MIA- and *Fmr1* KO vehicle-treated mice (Two-way ANOVA, Interaction F_1.41_ = 2.156, *p* = 0.1497, genotype effect F_1.41_ = 9.963, *p* = 0.0030 and treatment effect F_1.41_ = 14.18, *p* = 0.0005, WT vehicle vs WT MIA **p = 0.0029, WT vehicle vs *Fmr1* KO vehicle *p = 0.0206 in Tukey’s multiple comparisons test). **m** Representative western blots for FMRP and p-FMRP (Ser499). **n** FMRP levels decreased in WT MIA-treated adult animals (Mann-Whitney test, **p = 0.0068). **o** FMRP RNA-immunoprecipitation from hippocampal total extract followed by RT-qPCR revealed *Tsc2* mRNA as part of the FMRP complex. *H3* and *CamKIIa* mRNAs are negative and positive controls, respectively (Multiple unpaired t test, **p < 0.01, ***p < 0.001; WT and *Fmr1* KO *n* = 6). **p** *Tsc2* mRNA decreased in WT animals exposed to MIA as well as in *Fmr1* KO vehicle (Two-way ANOVA, Interaction F_1.31_ = 1.519, *p* = 0.2270, genotype effect F_1.31_ = 5.245, *p* = 0.0290 and treatment effect F_1.31_ = 4.856, *p* = 0.0351, WT vehicle vs WT MIA *p = 0.0403, WT vehicle vs *Fmr1* KO vehicle *p = 0.0403 in Holm-Šídák’s multiple comparisons test). **q** Representative western blots for TSC2 levels. **r** MIA affected TSC2 levels in WT adult animals, while no effect was observed in *Fmr1* KO MIA-treated mice (Two-way ANOVA, Interaction F_1.32_ = 13.61, *p* = 0.0008 and genotype effect F_1.32_ = 4.205, *p* = 0.0486, WT vehicle vs WT MIA **p = 0.0027, WT vehicle vs *Fmr1* KO vehicle **p = 0.0028 in Tukey’s multiple comparisons test; WT vehicle: *n* = 7–11; WT MIA: *n* = 7–13; *Fmr1* KO vehicle: *n* = 7–13; *Fmr1* KO MIA: *n* = 8–15). Data are represented as mean ± SEM. Total proteins were normalized to the average of Ponceau or Coomassie staining and vinculin. Phosphoproteins were normalized for the respective total protein levels. The molecular weight of each protein is indicated in kDa.

To investigate the molecular pathways underlying the core autistic-like symptoms observed in the two ASD models, we probed the mechanistic target of rapamycin (mTOR) pathway, which is dysregulated in FXS mice [69, 70]. We measured the protein levels and activity of mTOR, eukaryotic initiation factor 4E (eIF4E), eIF4E-binding proteins (4E-BPs), S6 kinase 1 (S6K1) and ribosomal protein S6 (rpS6) in the hippocampus of adult offspring of both WT and *Fmr1* KO from vehicle and MIA-treated groups (Fig. 4a–l and Supplementary Fig. S5a–e). The activity of mTOR was upregulated in both *Fmr1* KO vehicle mice and WT MIA-treated animals (Fig. 4a, b), as previously described [69, 71, 72], while Poly (I:C) treatment did not further affect mTOR activity in KO mice (Fig. 4a, b). Total mTOR levels remained unaffected (Supplementary Fig. S5a). Furthermore, *Fmr1* KO mice displayed increased activity of eIF4E independent of the received treatment (Fig. 4c, d), with no changes in the total levels of eIF4E compared to WT mice (Supplementary Fig. S5b). Interestingly, we observed reduced levels of 4E-BP1 activity in *Fmr1* KO mice and decreased total 4E-BP1 levels following MIA exposure (Fig. 4e, f and Supplementary Fig. S5c). The levels of 4E-BP2 were also reduced in both WT MIA and *Fmr1* KO vehicle-treated animals, while MIA exposure did not affect 4E-BP2 expression in *Fmr1* KO mice (Fig. 4g, h).

To further corroborate the hyperactivity of mTOR following the MIA condition, we examined its downstream effector, the S6K1. Our findings revealed an increased activity of both S6K1 (Fig. 4i, j) and its target rpS6 (Fig. 4k, l) in WT MIA-treated animals and in *Fmr1* KO vehicle-treated mice, while total protein levels did not change (Supplementary Fig. S5d, e). Noteworthy, MIA-treatment in *Fmr1* KO animals did not exacerbate the pre-existing altered activity of S6K1 and rpS6 (Fig. 4i–l), supporting the lack of synergistic effects of MIA in FXS. These results suggest that an immune insult in WT mice *in utero* affects both cap-dependent protein translation and ribosome biogenesis.

Because the lack of FMRP occludes the MIA effect on core autistic-like behaviors, we hypothesized that FMRP might be the common downstream effector in both the genetic and the environmental models of ASD. To test this possibility, we measured FMRP total levels and activity in the hippocampus of adult WT offspring following MIA exposure. Phosphorylation of FMRP at Ser499 (Supplementary Fig. S5f, g) did not change in both groups. Interestingly, we found a 20% decrease of FMRP levels specifically in the hippocampus of MIA-treated WT animals compared to vehicle-treated mice (Fig. 4m, n) while *Fmr1* mRNA levels were similar in both groups (Supplementary Fig. S5h). Unlike the MIA-treated animals, PIA did not affect FMRP levels in WT mice (Supplementary Fig. S5i, j). Of note, FMRP reduced levels were specific to the hippocampus, as no changes were observed in the cortex (Supplementary Fig. S5k, l) or cerebellum (Supplementary Fig. S5m, n). We next investigated whether the decreased FMRP levels were due to an impaired *Fmr1* mRNA translation. Polysome-mRNPs distribution analysis in hippocampus [41] showed a reduced association of *Fmr1* mRNA with actively translating polysomes in MIA-treated mice compared to controls, with no changes in *β-actin* mRNA distribution between polysomes and mRNPs (Supplementary Fig. S6a, b). This is consistent with the documented effect of MIA on pathways related to protein synthesis [73]. Additionally, since it was shown that mGluR activation triggers FMRP degradation [74], we assessed FMRP ubiquitination in the hippocampus of MIA-treated offspring and found increased ubiquitination compared to controls (Supplementary Fig. S6c). This indicates that MIA impacts both *Fmr1* translational efficiency and FMRP protein stability.

Overall, our findings suggest that reduced FMRP levels in MIA-treated animals may lead to mTOR hyperactivation. Based on previous studies indicating that *Tsc2* mRNA, encoding for a protein inhibiting mTOR, is a putative target of FMRP [30, 75–77], we performed an RNA-immunoprecipitation from the hippocampus of WT animals and found that *Tsc2* mRNA is indeed part of the FMRP complex (Fig. 4o). Notably, *Tsc2* mRNA (Fig. 4p) as well as TSC2 protein levels (Fig. 4q, r) were downregulated in KO vehicle-treated and in WT MIA-treated mice (Fig. 4p–r). These findings support the hypothesis that FMRP downregulation leads to mTOR hyperactivation through the regulation of *Tsc2* mRNA metabolism.

## Discussion

**Fig. 5:**
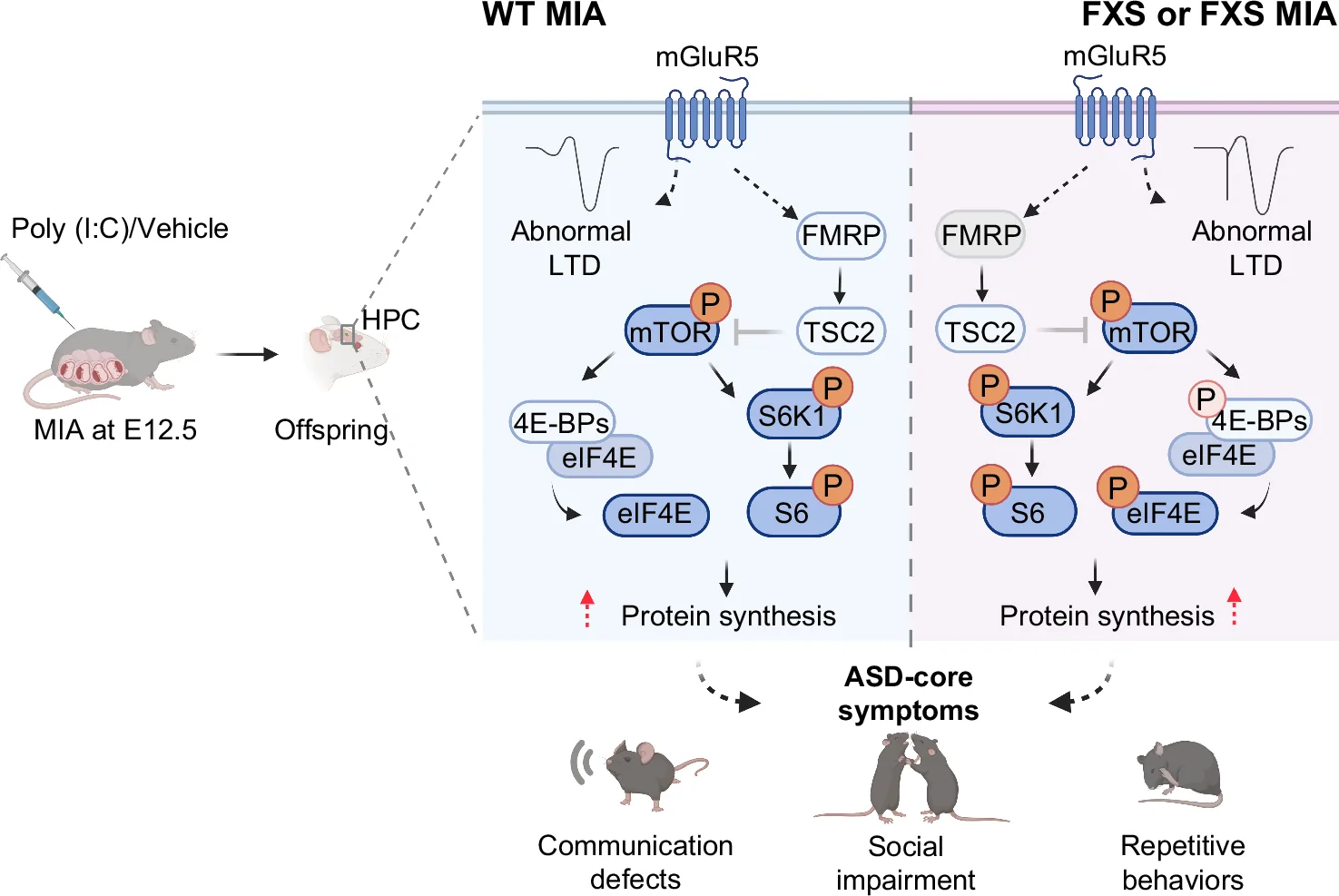
*Fmr1* mutation and MIA exposure lead to ASD-like behaviors and impairments in the mGluR1/5-mTOR pathway. Proposed mechanism by which the genetic FXS and the environmental MIA conditions trigger ASD-like behaviors. FMRP modulates mTOR activity via TSC2, since *Tsc2* mRNA is part of the FMRP complex. Mechanistically, MIA treatment in WT mice downregulates FMRP levels specifically in the hippocampus where it also upregulates mTOR activity, enhances S6K1 and rpS6 activities, while decreasing 4E-BPs and TSC2 levels. The dysregulation of these molecules suggest that MIA triggers exaggerated protein synthesis. mGluR1/5 signaling is impaired following MIA exposure and results in a shift towards LTP through an FMRP-dependent mechanism. The *Fmr1* mutation alone (or combined with MIA) leads to similar molecular mechanisms, including increased mTOR, S6K1, rpS6 and eIF4E activities while it reduces 4E-BPs and TSC2 levels, resulting in exaggerated protein synthesis. Dashed lines represent indirect regulation. Red dashed lines suggest an increased protein synthesis in both conditions that converge into ASD-like phenotypes. Dark blue and dark orange indicate proteins and phosphoproteins that increased in WT MIA, FXS or FXS MIA conditions, respectively. Light blue and light orange indicate proteins and phosphoproteins that were decreased under WT MIA, FXS or FXS MIA conditions, respectively. HPC = hippocampus.

Our study shows that both the *Fmr1* mutation and exposure to MIA led to similar core autistic traits, including impaired social communication and interaction, and repetitive behaviors, but not to associated comorbidities, such as hyperactivity and anxiety. Mechanistically, MIA treatment in WT mice downregulates hippocampal FMRP levels, reduces TSC2 levels and leads to mTOR overactivation. The *Fmr1* mutation alone enhances the mTOR signaling compared to WT vehicle-treated mice. In addition, MIA disrupts the mGluR1/5-LTD pathway in WT animals, resulting in a shift towards robust LTP. These results could explain the hyperactivation of protein translation documented in both conditions. Altogether, our findings show a common hyperactivation of the mGluR1/5-mTOR pathway, downregulation of FMRP and core autistic-like behavior in both genetic (FXS) and environmental (MIA) models of ASD (Fig. 5), suggesting the presence of shared molecular mechanisms.

### *Fmr1* mutation occludes MIA effects on ASD-like behaviors

ASD and FXS heterogeneity may arise from gene-environment interactions [25, 78]. Compelling evidence supports this interaction, although heterogenous results depending on the genetic background and nature of the insult were reported [79]. Likewise, studies on MIA models show different results on ASD-like phenotypes, possibly due to different Poly (I:C) formulations and age of the offspring [36, 80].

We found no synergistic effect of MIA in the *Fmr1* genetic model on core symptoms of ASD, in agreement with previous studies [81, 82], suggesting that the absence of FMRP occludes - in our tested conditions - the effects caused by the MIA. As FXS is an X-linked condition and ASD is more prevalent in males than females, with a male-to-female ratio of 4:1, our study focused on potential impact of MIA in the male offspring. While we did not investigate the specific effect of MIA on the female progeny, a recent study demonstrated that an insult such the unpredictable chronic stress during pregnancy does not exacerbate the behavioral phenotypes in *Fmr1* *Het* female progeny [82]. Recent findings indicate that FMRP orchestrates the immune response in cancer cells [83]. Importantly, while FXS has been linked to a perturbed immune system in rodents and humans [84–88], human clinical data on the peripheral inflammatory biomarkers have provided controversial results [88]. In agreement with previous observations, we did not detect differences in the cytokine levels between pregnant WT and *Fmr1 Het* female animals [89], suggesting that the absence of FMRP does not affect the acute phase of the immune response. Further research is needed to understand a possible contribution of FMRP to the different aspects of the immune response including viral infections.

### Hippocampal FMRP is a crucial hub linking MIA and FXS

While preclinical and clinical studies support the hypothesis of a MIA contribution to NDDs [90], the molecular mechanisms underlying neuronal abnormalities in offspring of mothers who experienced infections during pregnancy have yet to be elucidated. Data from gene expression studies on models of MIA revealed that ASD- and SCZ-associated genes are among the most dysregulated upon MIA [91]. Growing evidence suggests that MIA influences key processes of neurodevelopment, including microglial and immune system development [92–94] as well as ribosome biogenesis and protein homeostasis in the fetal brain [73].

We show that MIA, but not PIA, leads to a long-lasting downregulation of FMRP in the hippocampus of the adult offspring, with no effect in the cortex or cerebellum. To our knowledge, this is the first study showing a downregulation of FMRP upon MIA, indicating the presence of an FMRP-dependent vulnerable pathway hippocampus-specific. Our findings suggest that FMRP may be a key player for proper hippocampal development and function. Besides learning and memory, the hippocampus is also involved in core ASD-like behaviors, including social interactions [95]. Notably, children with ASD display dysregulated immune system signaling in the hippocampus [96], suggesting that the hippocampus might be vulnerable to immune-mediated changes. In addition, a recent work revealed that loss of FMRP leads to age-dependent alterations in hippocampal protein expression, with different proteome profile between early development (P7) and adulthood (P90) [97], that could partially explain why no changes were observed in the FMRP levels upon PIA.

MIA exposure leads to reduced expression of a subset of FMRP target mRNAs in the fetal brain [34] and recent publications have reported a reduction of FMRP levels in subjects with ASD [98, 99]. Further investigations are required to understand how MIA exposure leads to the downregulation of FMRP. Here, we show that *Fmr1* mRNA levels or FMRP phosphorylation at S499 are not affected in the offspring of MIA. Importantly, we observe that MIA modulates the efficiency of *Fmr1* mRNA translation, suggesting that immune challenges *in utero* affect proper FMRP synthesis during critical periods of brain development. In addition, our findings show that FMRP degradation via ubiquitination is impaired following MIA. Of note, FMRP degradation requires mGluR activation, and its degradation is also increased in an mouse model of ASD with the TSC2-deficiency [74, 100]. Accordingly, our data demonstrate that MIA disrupts the mGluR5 signaling, which might in turn stimulate a proteasomal degradation of FMRP. As FMRP regulates mRNA translation [31], we hypothesize that a dysregulation of protein synthesis might occur in both genetic and environmental mouse models of ASD.

Overall, our converging evidence reveals how MIA affects the synthesis and stability of FMRP, providing novel mechanistic insights into how an immune insult might contribute to the development of ASD-like features.

### The mTOR-FMRP pathway contributes to MIA and FXS associated phenotypes

Besides the downregulation of FMRP, we show that both MIA and the FXS conditions lead to the hyperactivation of mTOR, as previously described [25, 34, 71, 101] and potentially increased rate of translation through the dysregulation of distinct components of the mTOR signaling pathway. mTOR plays a pivotal role in the regulation of translation, cell growth as well as the immune response [102]. mTOR is dysregulated in ASD [103–108], with a score 1S (high confidence, syndromic) in the SFARI database (https://gene.sfari.org/database/human-gene/MTOR) [108]. Moreover, mTOR has been suggested to contribute to ASD-like traits following MIA [71, 72]. The combination of  *TSC1/2* mutation with maternal or early postnatal immune activation leads to a synergistic effect that in mice affects social behavior and social memory [109, 110]. Noteworthy, we found that, in the hippocampus, the mRNA of *Tsc2* is part of the FMRP complex, and TSC2 protein levels decrease in both WT MIA and *Fmr1* KO vehicle-treated animals, suggesting that the downregulation of FMRP  promotes the hyperactivity of mTOR and abnormal mGluR5-LTD via TSC2.

The mTOR pathway is the downstream effector of different receptors, including the mGluR5 [111] and it has been suggested that mGluR5-LTD requires the activation of mTOR in hippocampal synapses [111, 112]. LTD pathways have been found to be enriched in genes that are overexpressed following MIA [113]. We show that MIA impairs mGluR5-LTD, resulting in a robust induction of LTP in hippocampal synapses of the adult offspring, like previous findings in the LPS model of MIA [35]. Importantly, combining the *Fmr1* mutation with exposure to MIA does not lead to the LTD-to-LTP switch observed in slices from WT MIA-treated animals. Moreover, exposure to inflammation during puberty (PIA) impairs mGluR5-LTD in WT mice but not in *Fmr1* KO mice. Our data indicate that an exposure to inflammation, during gestation (MIA) or puberty (PIA), disrupts the mGluR5-LTD in hippocampal synapses, promoting an LTD-to-LTP shift in WT animals but not in *Fmr1* KO animals, supporting the hypothesis of an occlusion effect in the absence of FMRP.

### The effects of immune activation are evident during a critical period of development

Importantly, the WT MIA-treated and PIA-treated animals exhibit a significant increase of LTP (35%) and (14%), respectively, compared to controls, consistent with a major change in the levels of FMRP occurring only in the MIA offspring. Because in mice the expression of FMRP is developmentally regulated, with high levels during embryogenesis and the early postnatal period, it is reasonable to hypothesize that the embryonic stage, characterized by high levels of FMRP, is the most vulnerable period to immune insults [114]. While our data demonstrate that MIA in WT mice leads to ASD-like behaviors, PIA does not induce any core autistic-like phenotype during adulthood, indicating that immune activation during a specific developmental window affects adult behavior. Moreover, PIA does not affect the expression of FMRP at adult stage in which FMRP expression is already much lower than during early development, and the brain has already been largely wired. Therefore, we could hypothesize that the abnormal LTD upon PIA is less FMRP-dependent and might involve a different molecular mechanism. Accordingly, treatment with LPS during adulthood does not affect social behavior in *Fmr1* KO mice [115], further supporting the hypothesis that the vulnerable period to immune challenge is during development.

The maternal immune activation may affect synaptogenesis during early post-natal development, thus formulating the concept of “immune-synaptopathy” [116]. Emerging evidence suggests that specific cytokines, which are physiological neuromodulators, play a role in behavioral phenotypes caused by MIA [12, 47, 117, 118]. MIA leads to the release of pro-inflammatory cytokines which can penetrate the fetal circulation or activate resident immune cells, ultimately affecting brain homeostasis [47, 119, 120]. Consistent with this role, exaggerated levels of maternal cytokines, such as IL-6, trigger behavioral and neuropathological changes in the offspring [117, 121].

In addition to the lack of additive effects on autistic-like traits in *Fmr1* KO PIA-treated mice, we show that an immune insult during puberty does not exacerbate sensorimotor gating deficits in *Fmr1* KO mice. In WT mice instead, Poly (I:C) treatment during adolescence reduces PPI, consistent with a previous work showing a deficit after treatment during very early postnatal stages [122]. While the relationship between FXS and SCZ is still unclear, emerging evidence indicates an association between the expression of FMRP and SCZ [30, 123, 124]. SCZ in the FXS condition is poorly documented in clinics, and only a case study has so far reported SCZ symptoms in an individual with FXS [125], therefore future clinical studies are necessary to further explore this possible link. Overall, our findings indicate that critical periods of brain development are especially vulnerable to immune insults, which can lead to significant long-lasting changes, such as the alteration of FMRP levels, ultimately affecting synaptic plasticity and behavioral outcomes. 

## Supplementary information

Hilal Rosina et al suppl material

## Data Availability

The data supporting this study are available from the corresponding author upon reasonable request.
