# Supplementary material for: Dysregulation of the mTOR-FMRP pathway and synaptic plasticity in an environmental model of ASD
Source: Mol Psychiatry. 2024 Nov 27;30(5):1937–51. doi: 10.1038/s41380-024-02805-0 (PMC12014490; doi:10.1038/s41380-024-02805-0)

Supplementary Information

Dysregulation of the mTOR-FMRP pathway and synaptic plasticity in an environmental model of ASD

Muna L. Hilal[#], Eleonora Rosina[#], Giorgia Pedini, Leonardo Restivo and Claudia Bagni*

SUPPLEMENTARY FIGURE LEGENDS

Supplementary Fig. 1. Maternal immune activation and the *Fmr1* mutation alter sociability. (**a**) Representative heatmaps of the social preference session. (**b**) Bar plots showing the time spent with the stranger mouse vs time spent with the inanimate object revealed preference towards the exploration of the stranger over the inanimate object for WT vehicle animals, while no preference was observed in *Fmr1* KO vehicle. WT MIA-treated mice do not display social preference, although *Fmr1* KO animals exposed to MIA present occluded effects (Three-way ANOVA test, stimulus effect $F_{1.51} = 3.35$, $p = 0.073$, stimulus x genotype x treatment, $F_{1.51} = 0.66$, $p = 0.418$). (**c**) Representative heatmaps of the social novelty session. (**d**) Bar plots showing the time spent with the stranger mouse vs time spent with the familiar animal revealed a treatment effect in WT animals, while social novelty was not affected in KO animals upon MIA (WT vehicle: n = 11 - 12; *Fmr1* KO vehicle: n = 14; WT MIA: n = 14 - 15; *Fmr1* KO MIA: n = 16; Three-way ANOVA test, stimulus effect $F_{1.53} = 28.93$, *** $p < 0.001$, stimulus x genotype x treatment, $F_{1.53} = 0.5922$, $p = 0.445$; Two-way ANOVA, Interaction $F_{3.106} = 0.8404$ $p = 0.4747$ time spent with stranger vs time spent with familiar ****$p < 0.0001$, WT vehicle: time with stranger vs time with familiar **$p = 0.0024$; KO vehicle: time with stranger vs time with familiar *$p = 0.0244$; KO MIA: time with stranger vs time with familiar * $p = 0.0470$ in Tukey's multiple comparisons test). Data are represented as mean ± SEM.

Supplementary Fig. 2. Maternal exposure to Poly (I:C) increases cytokine and chemokine levels in pregnant dams, with no inflammation in the adult offspring of MIA-treated females. (**a**) The weight gain following mating of *Fmr1 Het* females is measured to precisely detect pregnancy at E12.5, when Poly (I:C) *i.p* injection takes place. (**b**) Dose-response analysis. Serum levels of the proinflammatory cytokine IL-6 in pregnant WT female mice injected with increasing doses of Poly (I:C) show enhanced IL-6 levels in both 20 mg/kg and 40 mg/kg doses compared to WT vehicle-treated dams, with no further increase at the higher dose (40 mg/kg) (n = 6/group; Kruskal-Wallis test H(4) = 11.93, **$p = 0.0076$, WT vehicle vs WT 20 mg/kg **$p =$

0.0098, WT vehicle vs WT 40 mg/kg *p = 0.0341, Dunn's multiple comparisons test). (**c**) Maternal serum IL-17a levels increase upon Poly (I:C) injection in both pregnant WT (left) and *Fmr1 Het* (right) females (n = 6 technical replicates of 3 independent biological sample *per* group; Student's unpaired t-test). (**d, e, f**) Poly (I:C) injection enhances maternal serum IL-6 (**d**), RANTES (CCL5) (**e**) and TNF-α (**f**) levels in both pregnant WT (left) and *Fmr1 Het* (right) females (n = 6 technical replicates of 3 independent biological sample *per* group; Student's unpaired t-test, ****$p < 0.0001$). (**g**) Maternal care, measured as latency for pup retrieval at postnatal day 4 (P4), shows similar latencies between vehicle and Poly (I:C) injected dams (WT vehicle: n = 8; WT MIA: n = 8; Student's independent samples t-test). (**h**) Peripheral inflammation measured in the adult offspring shows no difference in IL-6 levels between groups (WT vehicle: n = 11; WT MIA: n = 14; *Fmr1* KO vehicle: n = 14; *Fmr1* KO MIA: n = 16; Two-way ANOVA test, Interaction $F_{1,.51}$ = 0.2914, p = 0.5917). Data are represented as mean ± SEM except panel (a) where the results are shown as mean ± SD.

Supplementary Fig. 3. PIA does not trigger ASD-like behaviors and sensorimotor gating defects. (**a**) Illustration of the experiment timeline. Poly (I:C) at 20mg/kg was administered to WT or *Fmr1* KO male mice at P35, and behavioral tests conducted in adulthood. The same cohort of animals, also used in Fig. 3g, was analyzed across the different behavioral tests. (**b**) Body weight change upon treatment shows comparable body weight loss in WT and *Fmr1* KO mice one day after injection (Two-way ANOVA, Interaction $F_{1.47}$ = 1.294, p = 0.2611 and treatment effect $F_{1.47}$ = 12.64, ***p = 0.0009, WT vehicle vs WT PIA *p = 0.0163 in Tukey's multiple comparisons test). (**c**) No change in body weight is observed six days after treatment with Poly (I:C) at P35 (Two-way ANOVA test, Interaction $F_{1.47}$ = 1.294, p = 0.2611 and treatment effect $F_{1.47}$ = 0.2453, p = 0.6227). (**d**) Hyperactivity measured in the open field test. *Fmr1* KO mice travel longer distances compared to WT mice independently of the treatment (Two-way ANOVA, Interaction $F_{1.28}$ = 0.1585, p = 0.6936 and genotype effect $F_{1.28}$ = 5.844, *p = 0.0224). (**e**) Novel object exploration test conducted in an open field arena shows no difference between groups (Two-way ANOVA test, Interaction $F_{1.28}$ = 0.7451, p = 0.3954 and genotype effect $F_{1.28}$ = 1.508, p = 0.2296). (**f**) WT vehicle-treated and WT PIA-treated animals display similar startle response, while *Fmr1* KO mice show decreased startle response which is not further affected by PIA (Two-way ANOVA test, Interaction $F_{1.33}$ = 0.6132, p = 0.4392 and genotype effect $F_{1.33}$ = 10.72, **p = 0.0025). (**g**) Average percentage of pre-pulse inhibition (PPI) in WT and *Fmr1* KO mice, vehicle- and PIA-treated, shows no difference in the sensorimotor gating between groups (Two-way ANOVA test, Interaction $F_{1.33}$ = 3.422, p = 0.0733). (**h**) Percentage of PPI for each pre-pulse intensity in WT and *Fmr1* KO mice, vehicle- and PIA-treated, shows an effect of the different intensities in the *Fmr1* KO and PIA conditions (Three-way ANOVA test, dB

$F_{3.132}$ = 24.84, ****$p < 0.0001$, genotype x treatment $F_{1.132}$ = 10.09, **$p = 0.0019$; WT vehicle: n = 7 - 12; WT PIA: n = 7 - 12; *Fmr1* KO vehicle: n = 8 - 15; *Fmr1* KO PIA: n = 7 – 14). Data are represented as mean ± SEM.

Supplementary Fig. 4. AIA does not cause ASD-like behaviors. (**a**) Illustration of the experiment timeline. C57BL/6J WT mice received a single dose of Poly (I:C) at 20mg/kg during early adulthood, specifically at P56, and behavioral tests were conducted two weeks later. The same cohort of animals was analyzed across the different behavioral tests. (**b**) Body weight change monitored over 6 days post injection of Poly (I:C). The figure shows a significant effect of Poly (I:C) in C57Bl/6J-treated mice over 6 days (Two-way ANOVA test, Interaction $F_{5.105}$ = 4.048, **$p = 0.0021$). (**c**) A significant loss in body weight in the Poly (I:C) group is detected after one day of injection compared to vehicle (Student's non-paired t-test; **$p < 0.01$). (**d**) Poly (I:C) treatment does not affect body weight changes six days after treatment with Poly (I:C) (Student's non-paired t-test; $p > 0.05$). (**e**) Repetitive behavior investigated using the marble burying test shows no effect of AIA treatment (Student's t-test for independent samples; $p > 0.05$). (**f**) Social preference in adult mice examined using the three-chamber test reveals no impact on social preference upon AIA treatment (Student's t-test for independent samples; $p > 0.05$). (**g**) Social novelty in the three-chamber test shows no difference between groups (Student's t-test for independent samples; $p > 0.05$). (**h**) Anxiety evaluated using the Elevated Plus Maze test shows no effect after adult immune activation (Student's t-test for independent samples; $p > 0.05$). (**i**) No effect of AIA treatment on hyperactivity measured in the open field test (Student's t-test for independent samples; $p > 0.05$). (**j**) Novel object exploration test conducted in an open field arena shows no difference between groups (Student's non paired t-test; $p > 0.05$; WT vehicle: n = 9 - 12; WT AIA: n = 9 – 12). (**k**) Percentage of modification in fEPSPs post LTD induction shows no difference in slices derived from vehicle- and AIA-treated animals following application of DHPG (Student's non paired t-test; $p > 0.05$; WT vehicle: n = 3; WT AIA: n = 4). Data are represented as mean ± SEM except panel (b) where the results are shown as mean ± SD.

Supplementary Fig. 5. MIA exposure does not alter hippocampal mTOR pathway protein levels or FMRP in other brain regions. (**a**) Levels of hippocampal mTOR are not affected by MIA exposure or *Fmr1* mutation in the adult offspring (Two-way ANOVA, Interaction $F_{1.48}$ = 0.007288, $p = 0.9323$). (**b**) *Fmr1* mutation and MIA exposure have no effect on total eIF4E levels (Two-way ANOVA, Interaction $F_{1.48}$ = 0.05857, p = 0.8098). (**c**) Levels of 4E-BP1 decrease following MIA exposure regardless of the genotype (Two-way ANOVA, Interaction $F_{1.48}$ = 0.6165, p = 0.4362 and treatment effect $F_{1.48}$ = 5.984, *$p = 0.0182$). (**d**) S6K1 total levels do not change between treatment or genotype groups (Two-way ANOVA, Interaction $F_{1.32}$ = 1.305, $p = 0.2618$). (**e**) rpS6 expression is not affected by MIA exposure or *Fmr1* mutation in the adult offspring (Two-way ANOVA,

Interaction $F_{1.41} = 0.6855$, $p = 0.4125$) (WT vehicle: n = 8 - 11; WT MIA: n = 10 - 13; *Fmr1* KO vehicle: n = 9 - 13; *Fmr1* KO MIA: n = 8 - 15). (**f**) Representative western blots illustrate p-FMRP protein levels in the adult mouse brain of WT and *Fmr1* KO mice. The production and specificity of the p-FMRP antibody are assessed using brain protein extracts prepared with a lysis buffer containing or not a phosphatase inhibitor. (**g**) MIA treatment has no effect on p-FMRP levels in WT animals (Mann-Whitney test). (**h**) MIA treatment has no effect on *Fmr1* mRNA in WT animals (Mann-Whitney test; WT vehicle: n = 10; WT MIA: n = 12 - 13). (**i**) Representative western blots of FMRP protein levels in WT Vehicle and WT PIA. (**j**) PIA treatment has no effect on hippocampal FMRP levels (WT vehicle: n = 6; WT PIA: n = 8; Mann-Whitney test). (**k**) Representative western blots show FMRP levels in the cortex from adult offspring of WT Vehicle, *Fmr1* KO Vehicle, WT MIA, and *Fmr1* KO MIA. (**l**) Levels of cortical FMRP are not affected by MIA exposure in the adult offspring (Mann Whitney test). (**m**) Representative western blots show FMRP levels in the cerebellum from adult offspring of WT Vehicle, *Fmr1* KO Vehicle, WT MIA, and *Fmr1* KO MIA. (**n**) FMRP expression upon MIA does not change in the cerebellum from the adult offspring (Mann Whitney test; WT vehicle: n = 11; WT MIA: n = 12 – 13). Total proteins were normalized to the average of Ponceau or Coomassie staining and vinculin. In each western blots, the molecular weight of each protein is indicated in kDa. Data are represented as mean ± SEM.

Supplementary Fig. 6. Maternal immune activation affects *Fmr1* mRNA translation and FMRP degradation. (**a**) Representative UV absorbance profile of Polysomes/mRNPs cosedimentation analysis; adsorbance at 254 nm is indicative of rRNA concentrations. (**b**) Analysis of the relative distribution of *Fmr1* and *β-actin* mRNAs, left and right bar plots respectively, on a polysome-mRNPs gradient corresponding to actively translating polysomes and silent mRNPs. *Fmr1* mRNA distribution on actively translating polysomes is reduced following exposure to MIA (n = 6/group; Two-way ANOVA, Interaction $F_{1.20} = 99.73$, $p < 0.0001$, *Fmr1* mRNA distribution on polysomes in WT vehicle-treated vs WT MIA-treated mice ****$p < 0.0001$, *Fmr1* mRNA distribution on mRNPs in vehicle animals vs MIA mice ****$p < 0.0001$ in Tukey's multiple comparisons test). (**c**) Hippocampi from WT vehicle and WT MIA-treated animals were processed using the Signal-Seeker Ubiquitination detection kit (Cytoskeleton, Cat. # BK161-S). Left, representative western blot shows FMRP protein levels from MIA and vehicle-treated animals (input (1/20), lanes 1 and 2, respectively) and FMRP protein recovered after Ubiquitin-IP and controls (control beads without ubiquitin binding domain) (lanes 3 and 4, ubiquitin-IP MIA and vehicle mice, respectively and lanes 5 and 6, ubiquitin-IP CTRL MIA and vehicle animals, respectively). Right, ubiquitinated FMRP is increased in MIA-exposed mice (n = 4/group; Multiple unpaired t test, *$p = 0.033$ input WT vehicle vs input WT MIA; *$p = 0.049$ IP-Ub WT vehicle vs IP-Ub WT MIA

in Holm-Šídák multiple comparisons test). Total FMRP levels or ubiquitinated FMRP levels were normalized to the average of Coomassie staining and β-actin. The molecular weight of each protein is indicated in kDa. Data are represented as mean ± SEM.

SUPPLEMENTARY MATERIALS AND METHODS

Animals

Male *Fmr1* knockout (KO) mice and wild-type (WT) littermates on a C57BL/6J background (The Jackson Laboratory, Bar Harbor, Maine, USA) were used for all the experiments. Mice were weaned on postnatal day 21 (P21), at which time mice were housed at a maximum of 5 *per* cage with same-sex littermates and kept on a 12-hour regular light/dark cycle (7:00-19:00), with food and water provided *ad libitum*. The experimenters were blind to both the genotype and the treatment group of the mice while collecting and analyzing the data. Unblinding was performed only after all experimental assessments were completed. The sample size was chosen to have the smallest number of cases that revealed a biologically meaningful effect.

For treatment allocation to pregnant dams, no specific randomization method was used. Experimenters simply ensured that weight gain was similar among all treated pregnant dams.

Ethical statement. All experiments were approved by the Veterinary Authorities (Canton Vaud, Switzerland and Rome, Italy) and carried out in accordance with the European Communities Council Directive of 24 November 1986 (86/609EEC) under the approved animal licenses (Vaud, Switzerland: VD3150 and Rome, Italy: 301/2024-PR).

Poly (I:C) injection

Fmr1 heterozygous (*Het*) females 8 -12 weeks old were mated overnight with WT males of the same age. The next morning (embryonic day 0.5, E0.5), vaginal plugs were checked, and the females weighed daily until E12.5 to confirm pregnancy. Poly (I:C) (P9582 Sigma-Aldrich - Merck, Darmstadt, Germany) was prepared on the day of the injection in sterile NaCl 0.9% at a final concentration of 40mg/ml. In total, 37 dams were randomly assigned to either 20mg/kg Poly (I:C) or vehicle and received an intraperitoneal (*i.p.)* injection at E12.5, a time-point equivalent to the beginning of the second trimester in humans [1, 2]. The time window E12.5 was chosen as it has been shown to induce behavioral phenotypes associated with autism in mice [3]. We have randomly selected *Fmr1 Het* pregnant females to receive either vehicle or Poly (I:C) injections, ensuring balance in sample sizes across treatment groups. Injected dams were weighed again to monitor for pregnancy loss on E13.5 and then left undisturbed until the delivery. Out of 26 Poly (I:C) injected dams, 11 had miscarriages the day after the injection, such a frequency agrees with previous studies [4], resulting in no surviving pups and the loss of the entire offspring. Male offspring and littermates analyzed in this study were derived from 15 Poly (I:C) and 11 vehicle-treated dams.

Pubertal Immune Activation (PIA) was induced in male animals during adolescence, at P35, while Adult Immune Activation (AIA) was modeled in adult male mice, at P56.

Pregnant dams treated with Poly (I:C) delivered litters with a balanced sex ratio compared to females exposed to vehicle, indicating no effect of *in utero* immune activation on the offspring sex. All pups remained with the mother until weaning age, e.g. P21. Male mice from a total of 7 distinct batches were used for subsequent experiments.

Behavioral assays

Ultrasonic vocalizations (USVs). Ultrasonic vocalizations were recorded in offspring littermates of vehicle- or MIA-treated mothers at postnatal day 4 (P4). Briefly, each individual pup was separated from its mother and placed in a sound-proof chamber containing a microphone suspended 15 centimeters above the pup (Avisoft SASLab Pro). USVs recording was performed at room temperature, which was maintained at 22 ± 1°C. Calls were recorded unfiltered for 3 minutes, after which each pup was returned to its home cage. Call detection was performed using an automatic threshold-based algorithm (amplitude threshold-based algorithm: -20 dB; hold time: 15 ms). Accuracy of call detection was verified by an experienced user blind to the experimental condition of the mouse.

Pup retrieval test. Single pups were removed from the home cage with the mother to register ultrasonic vocalizations (USVs) in the sound-proof chamber. The retrieval test was conducted upon the reintroduction of the tested pup in the home cage, a corner opposing the nest of its dam. Camera recordings allowed offline evaluation of the latency of the dam to retrieve each pup as an indicator of maternal care. All pups were retrieved within one minute of testing.

Open field test & novel object exploration. The test was performed as previously described in [5]. The apparatus consisted of a square grey PVC arena (45 × 45 × 45 cm) that was illuminated with dimmed white lights (11-12 lx in the center) coming from the floor of the room. Mice were introduced face to the wall of the arena and allowed to freely explore the arena for 10 minutes. A virtual center zone (15 × 15 cm), in the middle of the arena was included for the behavioral analysis as indicator for anxiety-like behavior. *Novel object exploration:* at the end of the 10 minutes, an unfamiliar object (glue stick tube) was introduced in the center of the open field and the mouse was left to explore the object for 5 minutes. The time spent exploring the object was assessed as an indicator for exploratory activity. A contact with the object was scored if the center of the mouse was detected at a distance shorter than 6 cm from the center of the object (Object diameter: 5 cm). The floor was cleaned between each trial to avoid olfactory clues. A video tracking system (Ethovision 11.0

XT, Noldus information systems, Wageningen, The Netherlands) recorded the total distance traveled (cm) and the time spent exploring each zone was calculated as indicators for locomotive activity and novel object exploration activity, respectively. Specifically, we calculated the exploration time expressed as percentage as previously described [2], using the following formula: time spent in zone/total time x 100.

Elevated plus maze test. The test was performed as previously described in [6]. The apparatus was made from grey PVC. The apparatus consisted of a central platform (6 × 6 cm) elevated from the ground (60 cm). Two opposing open (36 × 6 cm) and two opposing close (36 × 6 × 15 cm) arms stemmed from the central platform. Light conditions were maintained at 17-18 lx in the open arms, and 3–4 lx in the closed arms. At the beginning of the test, each animal was individually placed in the middle of the apparatus and was allowed to freely explore the apparatus for 5 minutes. Mice were tracked (Ethovision 11.0 XT, Noldus information systems, Wageningen, The Netherlands) to measure the time spent in the open and closed arms. Entrance in arms was scored when the mouse crossed the border zone with all four paws.

Marble burying test. Standard polycarbonate rat cages (26 x 48 x 20 cm) fitted with filter-top covers were used for the marble burying test. Light conditions were maintained at 30 lux measured from the center of the test cage. Fresh and unscented mouse bedding material was added to each cage to a depth of 5 cm. The bedding surface was leveled by inserting another cage of the same size onto the surface of the bedding. Twenty standard glass toy marbles were gently placed on the surface of the bedding in 5 rows of 4 marbles. Each animal was placed in one of the corners and was left undisturbed for 30 minutes. A marble is counted as buried if 2/3 of its size is under the bedding and the percentage of buried marbles was evaluated for each animal as an indicator of repetitive behavior [7].

Three chamber test. The equipment used for social preference and social novelty is comprised of a rectangular, three-chamber box (38.5 x 58 x 21 cm, Noldus information systems, Wageningen, The Netherlands). Each chamber is 19 x 38.5 cm, and the dividing walls contained an open middle section, which allows free access to each chamber. Two identical transparent containers with transparent rods and removable lids large enough to hold a single mouse were placed vertically inside the apparatus, one in each side chamber. Stranger mice were age- and gender-matched to test subjects and were habituated to the containers for at least 30 minutes the day before testing. For each trial, an object (a yellow plastic tube) was placed inside one of the containers while a stranger mouse was housed in the other container. The location of the object and mouse was pseudo-randomized across trials. Light conditions were maintained at 10 lx in the middle of the three-chamber box. Mouse position was tracked (Ethovision 11.0 XT, Noldus information systems,

Wageningen, The Netherlands) to measure the time spent in the interaction zone (Width x Height: 15 cm x 14 cm), defined as the areas closest to both the stranger mouse and the object.

The social preference and social novelty indexes were calculated as previously described [8–10]. Briefly, the social preference index (%) is the result of the time spent exploring the stranger mouse / time spent exploring the stranger + the time exploring the unanimated object x 100. The social novelty index (%) is the result of the time spent exploring the stranger mouse / time spent exploring the stranger + the time exploring the familiar mouse x 100.

Pre-pulse inhibition (PPI). The pre-pulse inhibition was performed as previously described [11]. Briefly, mice were placed individually into the chamber (Med Associates, Inc., St. Albans, VT, USA) and exposed to an acclimation period (5 minutes + white noise at 65 dB) followed by a three-block session. Blocks 1 and 3 consisted of five pulse-alone (white noise, 120 dB, 40 ms) trials each. Block 2 was consisted of 5 prepulse-pulse trials with a variable intertrial interval of 15 seconds (ranging from 12 to 30 s). The pre-pulse had variable intensities of 0, 3, 7, 11 and 19 dB above the background (white noise: 65, 68, 72, 76, 84 dB) and was followed (80 ms onset-onset delay) by a pulse sound (white noise, 120 dB, 40 ms). Each prepulse-pulse trial was presented 10 times in a pseudo-randomized sequence. The PPI % was calculated as follows: % PPI = 100 – {[(startle response for prepulse + pulse)/(startle response for pulse-alone)] x 100}, where the startle response for pulse-alone was measured as the average of pulse-alone trials of block 2.

Autistic score. We built a score that encompasses the analyzed core symptoms of ASD by averaging the results of the performed behavioral tests for each animal. Specifically, the “Autistic score” was calculated as previously described for anxiety traits [12, 13], adapted to core autistic traits as an algebraic sum of standardized scores from the social preference index, marble burying index and USV duration index. Standardization was obtained as follows: (x – lowest value of the entire cohort) / (max value of the entire cohort – min value) where x is the value of each animal. This procedure yields scores ranging from 0 to 1, with 1 indicating a strong autistic-like behavior.

Electrophysiology

Juvenile adult male littermate mice aged between 9 and 12 weeks were used for all electrophysiological recordings. Following decapitation, brains were removed from the skull and placed in cold artificial cerebrospinal fluid (aCSF) solution. The aCSF composition was as follows (in mM): 125 NaCl, 2.5 KCl, 1.25 NaH_2PO_4, 26 $NaHCO_3$ and 12 glucose, 2 $MgCl_2$ and 1 $CaCl_2$, pH 7.4 (295–305 mOsm/L). Coronal brain slices of 350 µm were obtained using a vibratome (VT1000S Leica Biosystems, Nussloch, Germany). The two

hemispheres containing each a unilateral dorsal hippocampus were then separated and placed in aCSF saturated with 95% O_2 and 5% CO_2 at 33°C for 30 minutes. Slices were next left at least 90 minutes at room temperature before being transferred into the recording chamber. Slices were transferred into the recording chamber, maintained at 31°C and perfused (2ml/min) with oxygenated aCSF containing 2 mM Ca^{2+}, 2 mM Mg^{2+} and 50 µM of picrotoxin (1128 Tocris Bioscience, Bristol, United Kingdom). Epileptiform activity was avoided by cutting between CA3 and CA1 regions of the hippocampus.

To record activity of CA3-CA1 synapses, we electrically stimulated Schaffer collateral fibers and recorded CA1 field excitatory postsynaptic potentials (fEPSPs). After a stable baseline for at least 20 minutes, 50 µM of (S)-3,5-Dihydroxyphenylglycine (DHPG) (0805 Tocris Bioscience, Bristol, United Kingdom) was applied to the bath to induce mGluR5-dependent LTD. The slope of fEPSP was monitored for 60 minutes after induction, and averages of the last 10 minutes were compared between the different groups as described in the statistical analysis. Following statistical analysis showing a = b, impaired mGluR5 LTD is reported. If Statistical analysis shows a > b, LTD induction is concluded while the opposite case (a < b) indicates an LTP induction.

Ubiquitination pull-down

Ubiquitination of FMRP was detected using a Signal-Seeker Ubiquitination Detection Kit (BK161-S, Cytoskeleton, Denver, USA) according to the manufacturer's instructions. Briefly, hippocampi were isolated from adult WT mice born from mothers exposed to either vehicle or Poly (I:C) and homogenized in Blast R™ Lysis Buffer supplemented with de-ubiquitination/SUMOylation inhibitor (N-ethylmaleimide and TPEN, NEM09BB) and protease inhibitor cocktail (P8340 Sigma-Aldrich - Merck, Darmstadt, Germany). Hippocampus homogenates were precleaned using a BlastR filter and further diluted with BlastR dilution buffer. The samples (1.0 mg) were incubated with either control beads or ubiquitination affinity beads for 2,5 h at 4 °C with gentle rocking. After washing the beads with BlastR-2 wash buffer 3 times for 5 minutes each at 4 °C, the beads were centrifuged, and the pellets were incubated in bead elution buffer for 5 minutes at room temperature. Eluates were collected in the spin columns, dithiothreitol was added to the samples, which were then boiled for 5 minutes before running SDS–PAGE and western blot analysis, performed as described below.

Western blots

After behavioral tests, animals were subjected to one week of rest before sacrifice. Different brain regions from the 4 different cohorts of animals analyzed in the behavioral assays were quickly dissected following decapitation and stored at -80°C until use for biochemical analysis. In addition, some biochemical experiments

were also repeated with additional 3 cohorts of mice (2 Poly (I:C)-exposed animals and 1 vehicle-treated mice). Samples were homogenized with a tissue grinder dounce (WHEATON® Dounce Tissue Grinder 1 mL, VWR, Radnor, Pennsylvania, USA) in 150 mM NaCl, 50 mM Tris-HCl pH 7.5, 1% Triton X-100, 1% sodium deoxycholate, 1 mM EDTA pH 8.0, 0.5 mM dithiothreitol, a protease inhibitor cocktail (P8340 Sigma-Aldrich - Merck, Darmstadt, Germany) and a phosSTOP-phosphatase inhibitor cocktail tablet (4906837001 Roche - Merck). Brain region lysates were incubated 10 minutes on ice and centrifuged for 10 minutes at maximum speed at 4°C. Protein concentration was measured using the Pierce™ BCA Protein Assay Kit (23225, Thermo Fisher Scientific, Waltham, Massachusetts, USA).

Proteins (20 µg) were separated on a 4-15% Mini-PROTEAN™ TGX Stain-Free™ Protein Gels (4568086 Bio-Rad, Hercules, California, USA) and blotted on a PVDF membrane (Roche-Merck). Membranes were incubated using the following primary antibodies: rabbit anti-FMRP (1:1000 rAMII, [14]) or rabbit anti-FMRP (1:1000, AB17722 Abcam, Cambridge, UK), rabbit anti-p-FMRP (Ser499) (1:500, homemade, see Fig. S5f), mouse anti-mTOR (1:1000, 4517 Cell Signaling Technology), rabbit anti-p-mTOR (Ser2448) (1:500, 2971 Cell Signaling Technology), mouse anti-eIF4E (1:1000, SC-271480 Santa Cruz Biotechnology, Dallas, Texas, USA), rabbit anti-p-eIF4E (Ser209) (1:500, 9741 Cell Signaling Technology), rabbit anti-4E-BP1 (1:1000, 9644 Cell Signaling Technology), rabbit anti-p-4E-BP1 (Thr37/46) (1:500, 2855 Cell Signaling Technology), mouse anti-4E-BP2 (1:500, MABS1865 Sigma-Aldrich – Merck), mouse anti-p-p70 S6 Kinase (p-S6K1) (Thr389) (1:500, 9206 Cell Signaling Technology), rabbit anti-p70 S6 Kinase (S6K1) (1:1000, 9202 Cell Signaling Technology), rabbit anti-p-S6 Ribosomal Protein (p-rpS6) (Ser235/236) (1:500, 2211 Cell Signaling Technology), mouse anti-S6 Ribosomal Protein (rpS6) (1:1000, 2317 Cell Signaling Technology), rabbit anti-Tuberin/TSC2 (1:1000, 4308 Cell Signaling Technology), mouse anti-Vinculin (1:2000, V9131 Sigma-Aldrich - Merck) and mouse anti-β-Actin (1:5000, A5441 Sigma-Aldrich – Merck, Darmstadt, Germany). The following secondary antibodies were used: anti-rabbit or anti-mouse IgG Dylight 800 (1:2500, SA5-35571 and 1:5000 SA5-35521, respectively, Invitrogen - Thermo Fisher Scientific) and anti-mouse or anti-rabbit IgG Dylight 680 (1:1000, 35518 and 35568 Invitrogen - Thermo Fisher Scientific), and anti-rabbit IgG or anti-mouse IgG, HRP-linked Antibody (1:5000, 7074S and 7076S Cell Signaling Technology, Danvers, Massachusetts, USA). Proteins were revealed using the Odyssey Infrared Imaging System (LI-COR Biosciences, Lincoln, Nebraska, USA) or an enhanced chemiluminescence kit (1705061 Clarity Western ECL Substrate or 1705062 Clarity Max Western ECL Substrate - Bio-Rad) and the imaging system LAS-4000 mini (GE HealthCare Technologies Inc., Chicago, Illinois, USA).

Two different antibodies have been used in this study: FMRP antibody rAMII (Fig. 4m) and FMRP antibody from Abcam (Fig. S5k-m). FMRP antibody rAMII also detects an unspecific band.

Total and phospho-protein levels were detected simultaneously on the same membranes using either differentially fluorescently tagged secondary antibodies or by stripping and re-blotting with a second primary antibody. Specifically, to the latter method, after incubation with antibodies against phosphoproteins, the membranes were stripped by incubation with Restore™ PLUS Western Blot Stripping Buffer (46430 Thermo Fisher Scientific, Waltham, Massachusetts, USA) according to manufacturer's instructions. After each stripping, the absence of signal was confirmed before incubating the membrane with the antibody against the total protein.

All phosphoproteins were normalized relative to the total protein on the same blot. Protein levels were normalized using the average of Ponceau red staining or Coomassie blue staining and Vinculin signal on the membranes. Signal quantification was performed using ImageQuant TL software.

Enzyme-Linked Immunosorbent Assay (ELISA)

After 3 hours of vehicle or Poly (I:C) administration, blood was collected in heparin-coated tubes either from the submandibular vein or following rapid decapitation. Heparinized serum was isolated by centrifugation at 10000 *x* g for 4 minutes at 4°C. All samples were stored at −80 °C until further analysis. Interleukin 6 (IL-6) and Interleukin 17a (IL-17a) were measured using an ELISA kit (IL-6: ADI-900-045, Enzo Life Sciences, Ann Arbor, MI, USA; IL-17a: RAB0263 Sigma-Aldrich - Merck) according to the manufacturer's instructions.

Quantification of Serum Cytokine Levels using a Luminex Platform

After 3 hours of vehicle or Poly (I:C) administration, blood was collected in heparin-coated tubes either from the submandibular vein or following rapid decapitation. Blood was allowed to clot for at least 30 minutes and then centrifuged at 1000 *x* g for 10 minutes. Heparinized serum was collected and stored at −80 °C until further analysis. Mouse cytokine levels from blood serum were assessed at the ISO 9001:2015-certified Labospace Srl laboratory (Labospace, Milan, Italy). The following cytokines and chemokines were simultaneously detected in 30 µL of samples two-folds diluted: Interleukin 6 (IL-6), Regulated upon Activation Normal T cell Expressed and Secreted (RANTES, or C–C motif Chemokine ligand 5, CCL5) and Tumor Necrosis Factor α (TNF-α). The MILLIPLEX® Mouse Cytokine/Chemokine Magnetic Bead Custom-made panel (MCYTOMAG-70K, Millipore Sigma, Merck) was used according to the manufacturer's protocol. The multiplex panel simultaneously detect IL-6, RANTES (CCL5) and TNF-α via specifically mixed antibody-coated magnetic

beads. Plate was read with FLEXMAP 3D (SN: FM3DD19140021) and analyzed by Bio Plex 6.2 Software. Median fluorescent intensity (MFI) data were obtained for further analysis. Data in the scatter plots represent the mean of two technical replicates.

Polysomes-mRNPs analysis

The hippocampi from adult WT mice (P60), born from pregnant females treated with either vehicle or Poly (I:C), were lysed as previously described [15, 16]. The supernatant was loaded onto a 15–50% (w/v) sucrose gradient and sedimented by centrifugation at 4 °C for 150 minutes at 37,000 rpm in a Beckman SW41 rotor (Fullerton, CA, USA). Each gradient was collected into 10 fractions (1–6 = polysomes; 7–10 = mRNPs) while reading the absorbance at 254 nm, followed by the addition of 50 pg of spike-in control (luciferase control RNA, L456A, Promega, Madison, Wisconsin, USA). Total RNA was extracted from two separate pools: from polysomes and from mRNPs fractions. The RNA was precipitated, and its quality was assessed using gel electrophoresis and spectrophotometry (ND-1000 spectrophotometer, Nanodrop Technology, Thermo Fisher Scientific). The mRNAs of interest (*Fmr1*, *β-Actin* and *luciferase*) were quantified by RT-qPCR as described below.

FMRP RNA immunoprecipitation (RIP)

FMRP RNA immunoprecipitation was performed as previously described [17]. Briefly, hippocampi were isolated from adult WT and *Fmr1* KO mice and homogenized in ice-cold lysis buffer (250 mM NaCl, 20 mM Tris-HCl pH 7.4, 10 mM MgCl2, 1% Triton X-100, 10 ml/ml Protease inhibitor cocktail (P8340 - Sigma-Aldrich, Merck), 40 U/ml RNaseOUT™ Recombinant Ribonuclease Inhibitor (10777019 Invitrogen - Thermo Fisher Scientific) and centrifuged at 12,000 rpm for 10 minutes at 4°C. The supernatant was collected and incubated with Dynabeads™ Protein G (10003D Invitrogen - Thermo Fisher Scientific) coated with specific anti-FMRP antibody [18] in presence of 1% bovine serum albumin (BSA) for 90 minutes at 4°C. The beads were then washed with 250 mM NaCl, 20 mM Tris-HCl pH 7.4, 10 mM $MgCl_2$, 0.1% Triton X-100. The RNA from the immunoprecipitated eluate as well as a portion of initial lysate (input) was extracted with TRIzol according to the manufacturer's protocol (15596018 Invitrogen - Thermo Fisher Scientific, Waltham, Massachusetts, USA).

RNA purification and RT-qPCR

Total RNA from 50 µg brain lysate was isolated using 1 ml TRIzol™ Reagent (15596018 Invitrogen - Thermo Fisher Scientific, Waltham, Massachusetts, USA) according to the manufacturer's instructions. Following RNA

purification, RNA concentration was assessed with the NanoDrop™ Spectrophotometer (Thermo Fisher Scientific). First-strand synthesis was generated using 1µg of total RNA, 500 ng/ml p(dN)6 and 200 U/µl of the SuperScript™ III Reverse Transcriptase (18080044 Invitrogen - Thermo Fisher Scientific) or 200 U/ml M-MLV Reverse Transcriptase (28025013 Invitrogen - Thermo Fisher Scientific), buffer 5× M-MLV reaction buffer, RNase OUT and dNTPs, according to manufacturer's instructions. cDNA was diluted 1:20, 5 µl of cDNA was used and qPCR was performed using the LightCycler® 480 SYBR Green I Master (04707516001 Roche Applied Science, Penzberg, Germany) or the SsoAdvanced™ Universal SYBR® Green Supermix (1725271 - Bio-Rad, Hercules, California, USA) on StepOnePlus Real-Time PCR machine (Applied Biosystems - Thermo Fisher Scientific). For each gene, the primer pairs were designed using the NCBI primer Blast algorithm (http://www.ncbi.nlm.nih.gov/tools/primer-blast). Two technical replicates for each biological replicate were assessed. The mRNA expression level for each gene of interest was determined relative to a normalization factor (i.e., the average of the reference genes) using the $2^{-\Delta\Delta Ct}$ method [19]. The analysis of specific enriched mRNAs was normalized to *Hprt1* as control RNA and then calculated as the ratio of mRNAs found in the RIP-FMRP WT to the RIP-FMRP KO. Primers used:

Fmr1	forward:	5’-GCAGGGGGACCCCAGAAACCT-3’
Fmr1	reverse:	5’-ACAGTGGCATTAGCGATGCTGTC-3’
Actb	forward:	5’-CGTCCACCCGCGAGCACA-3’
Actb	reverse:	5’-TCCATGGCGAACTGGTGGC-3’
Cyp46a1	forward:	5’-TGGACTTGGCCTTCAGCCGC-3’
Cyp46a1	reverse:	5’-CCAGCTGCTCCGCCTTCTCG-3’
Gusb	forward:	5’-GTTGTGATGTGGTCTGTGGC-3’
Gusb	reverse:	5’-GGGCTTTGGTGTGGGTGAT-3’
H3	forward:	5’-CTTCCAGCGTCGCCTCGGTC-3’
H3	reverse:	5’-AGCGGTCTGCTTGGTTCGGG-3’
CamkIIa	forward:	5’-GTGCTGGCTGGTCAGGAGTATGC-3’
CamkIIa	reverse:	5’-CTTCAACAAGCGGCAGATGCGGG-3’
Hprt1	forward:	5’- CAGCCCCAAAATGGTTAAGGTTGC-3’
Hprt1	reverse:	5’- TCCAACAAAGTCTGGCCTGTATCC-3’
Tsc2	forward:	5’-AGCACGGAGCACCAGTCTTA-3’
Tsc2	reverse:	5’-CATCAGCCTGGGCCATAGAG-3’

Statistics

Statistical analyses were performed with GraphPad Prism software (Version 10.1.0 (264), San Diego, California, USA). The statistical tests used are listed in the Figure legends. Groups showing similar variance were subjected to the statistical comparisons. All data were tested for normality of their distribution to decide the appropriate statistical test. Unpaired two-tailed Student's t-tests or non-parametric two-tailed Mann-Whitney U-tests were used for comparisons between two groups for normal and non-normal distributed data, respectively. Multiple-t-test with Holm-Sidak multiple comparison correction was used for the immunoprecipitation experiments (namely FMRP RIP and Ubiquitination pull-down). When comparing more than two independent groups, one-way Analysis of Variance (ANOVA) test or Kruskal-Wallis test were used for non-normally distributed data. D'Agostino & Pearson normality test, Two-way ANOVA or Three-way Repeated Measures ANOVA with Bonferroni's or Holm-Sidak's or Tukey's multiple comparison tests were used for comparisons of three or more groups. One-sample t test was employed to assess whether the observed data deviated significantly from a chance level of 50%. Outliers were calculated using the Grubbs' outlier test and were excluded from the statistical analysis.

For electrophysiology experiments, averages of the last 10 minutes of fEPSP were compared among the different groups using the Two-way ANOVA, or to baseline 10 minutes prior to bath application of DHPG. The non-parametric Wilcoxon matched-pairs signed-rank test was employed. For all analyses, p values < 0.05 were considered significant and annotated as follows: *$p < 0.05$, **$p < 0.01$, ***$p < 0.001$, ****$p < 0.0001$. Results were presented as mean ± standard error of the mean (SEM) or mean ± standard deviation (SD).

REFERENCES

1. Chow K-H, Yan Z, Wu W-L. Induction of Maternal Immune Activation in Mice at Mid-gestation Stage with Viral Mimic Poly(I:C). J Vis Exp. 2016:e53643.
2. Ji-Xu A, Vincent A. Maternal Immunity in Autism Spectrum Disorders: Questions of Causality, Validity, and Specificity. J Clin Med. 2020;9.
3. Lammert CR, Lukens JR. Modeling Autism-Related Disorders in Mice with Maternal Immune Activation (MIA). Methods Mol Biol. 2019;1960:227–236.
4. Mueller FS, Richetto J, Hayes LN, Zambon A, Pollak DD, Sawa A, et al. Influence of poly(I:C) variability on thermoregulation, immune responses and pregnancy outcomes in mouse models of maternal immune activation. Brain Behav Immun. 2019;80:406–418.
5. Seibenhener ML, Wooten MC. Use of the Open Field Maze to measure locomotor and anxiety-like behavior in mice. J Vis Exp. 2015:e52434.
6. Walf AA, Frye CA. The use of the elevated plus maze as an assay of anxiety-related behavior in rodents. Nat Protoc. 2007;2:322–328.
7. Angoa-Pérez M, Kane MJ, Briggs DI, Francescutti DM, Kuhn DM. Marble burying and nestlet shredding as tests of repetitive, compulsive-like behaviors in mice. J Vis Exp. 2013:50978.
8. Nygaard KR, Maloney SE, Dougherty JD. Erroneous inference based on a lack of preference within one group: Autism, mice, and the social approach task. Autism Res. 2019;12:1171–1183.
9. Rein B, Ma K, Yan Z. A standardized social preference protocol for measuring social deficits in mouse models of autism. Nat Protoc. 2020;15:3464–3477.
10. Ma B, Shan X, Yu J, Zhu T, Li R, Lv H, et al. Social deficits via dysregulated Rac1-dependent excitability control of prefrontal cortical neurons and increased GABA/glutamate ratios. Cell Rep. 2022;41:111722.
11. Domínguez-Iturza N, Lo AC, Shah D, Armendáriz M, Vannelli A, Mercaldo V, et al. The autism- and schizophrenia-associated protein CYFIP1 regulates bilateral brain connectivity and behaviour. Nat Commun. 2019;10:1–13.
12. Bosch-Bouju C, Larrieu T, Linders L, Manzoni OJ, Layé S. Endocannabinoid-Mediated Plasticity in Nucleus Accumbens Controls Vulnerability to Anxiety after Social Defeat Stress. Cell Rep. 2016;16:1237–1242.
13. Larrieu T, Cherix A, Duque A, Rodrigues J, Lei H, Gruetter R, et al. Hierarchical Status Predicts Behavioral Vulnerability and Nucleus Accumbens Metabolic Profile Following Chronic Social Defeat Stress. Curr Biol. 2017;27:2202-2210.e4.
14. Ferrari F, Mercaldo V, Piccoli G, Sala C, Cannata S, Achsel T, et al. The fragile X mental retardation protein-RNP granules show an mGluR-dependent localization in the post-synaptic spines. Molecular and Cellular Neuroscience. 2007;34:343–354.
15. Zalfa F, Giorgi M, Primerano B, Moro A, Di Penta A, Reis S, et al. The Fragile X Syndrome Protein FMRP Associates with BC1 RNA and Regulates the Translation of Specific mRNAs at Synapses. Cell. 2003;112:317–327.
16. Napoli I, Mercaldo V, Boyl PP, Eleuteri B, Zalfa F, De Rubeis S, et al. The fragile X syndrome protein represses activity-dependent translation through CYFIP1, a new 4E-BP. Cell. 2008;134:1042–1054.
17. Mercaldo V, Vidimova B, Gastaldo D, Fernández E, Lo AC, Cencelli G, et al. Altered striatal actin dynamics drives behavioral inflexibility in a mouse model of fragile X syndrome. Neuron. 2023;111:1760-1775.e8.
18. Pedini G, Buccarelli M, Bianchi F, Pacini L, Cencelli G, D'Alessandris QG, et al. FMRP modulates the Wnt signalling pathway in glioblastoma. Cell Death Dis. 2022;13:719.
19. Fernández E, Gennaro E, Pirozzi F, Baldo C, Forzano F, Turolla L, et al. FXS-Like Phenotype in Two Unrelated Patients Carrying a Methylated Premutation of the FMR1 Gene. Front Genet. 2018;9:442.

Supplementary Figure 1

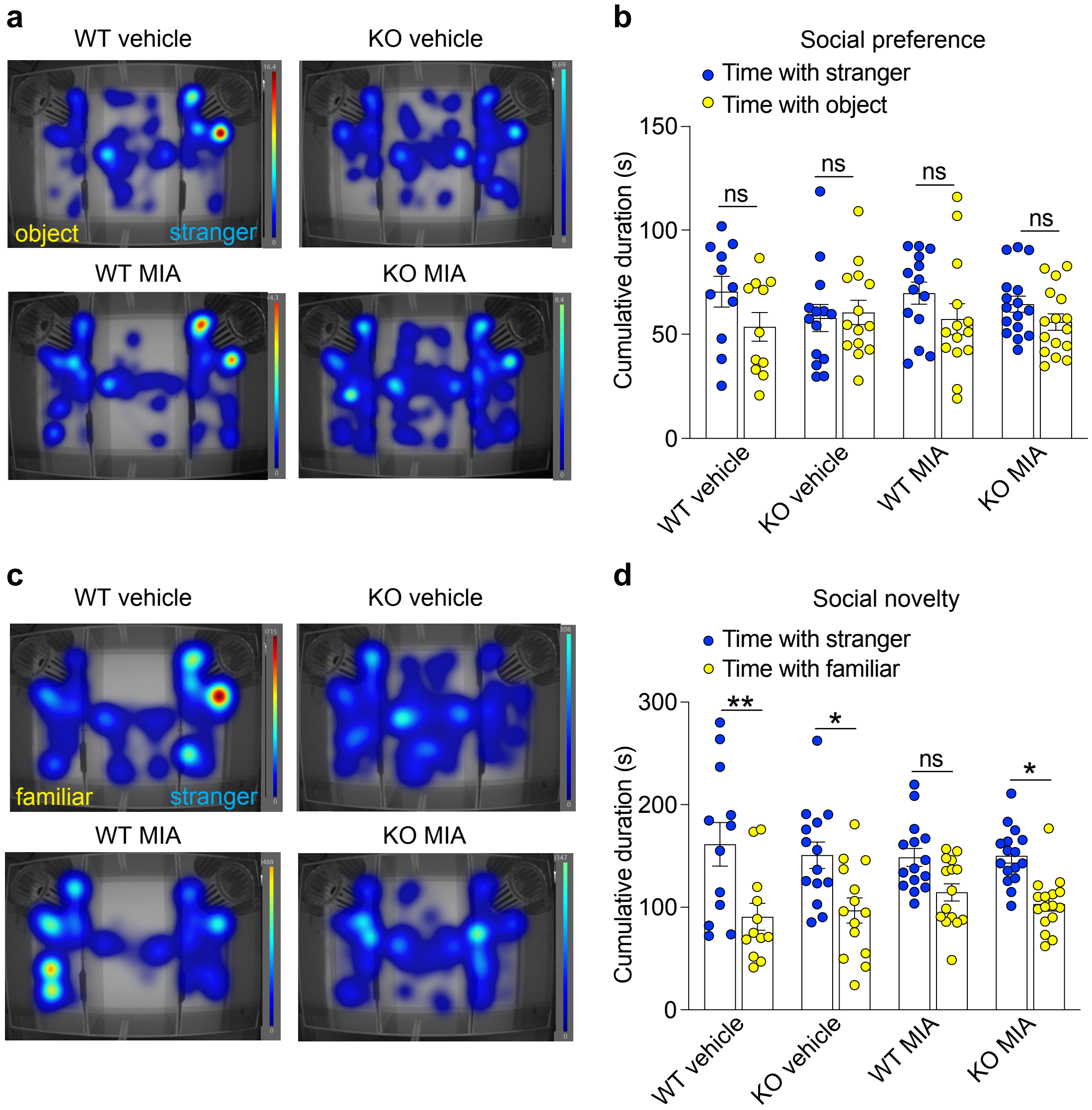

Supplementary Figure 2

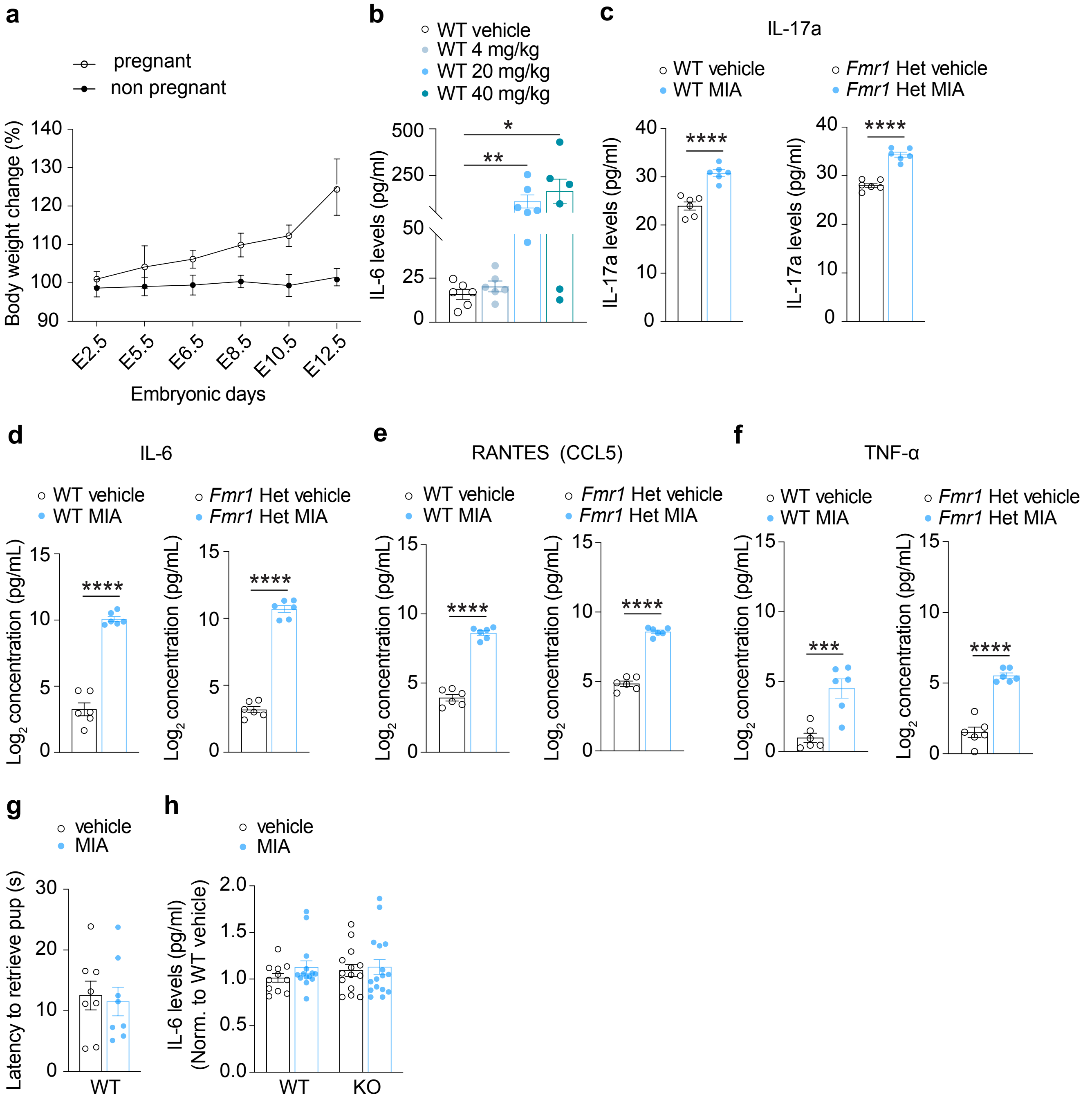

Supplementary Figure 3

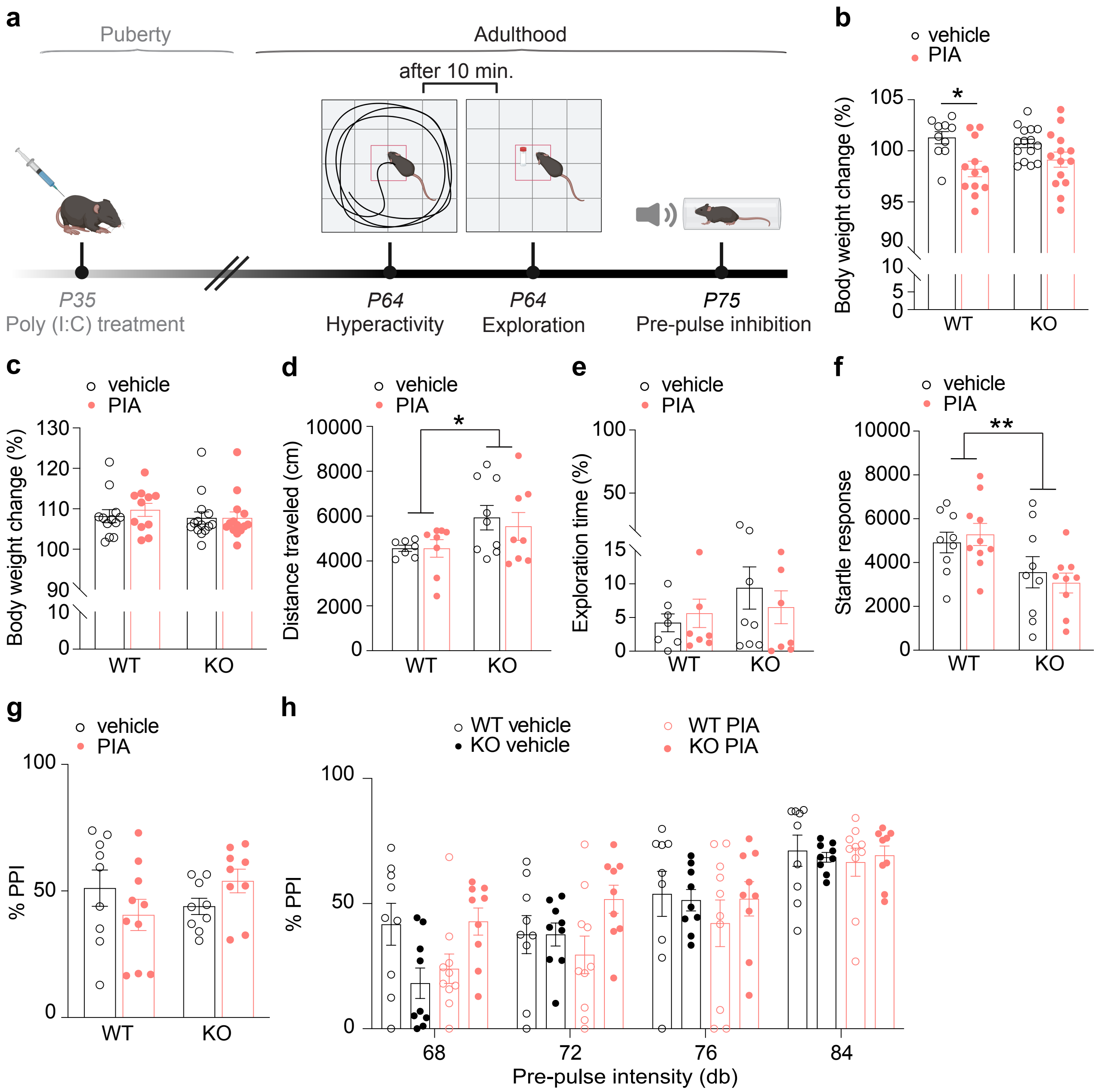

Supplementary Figure 4

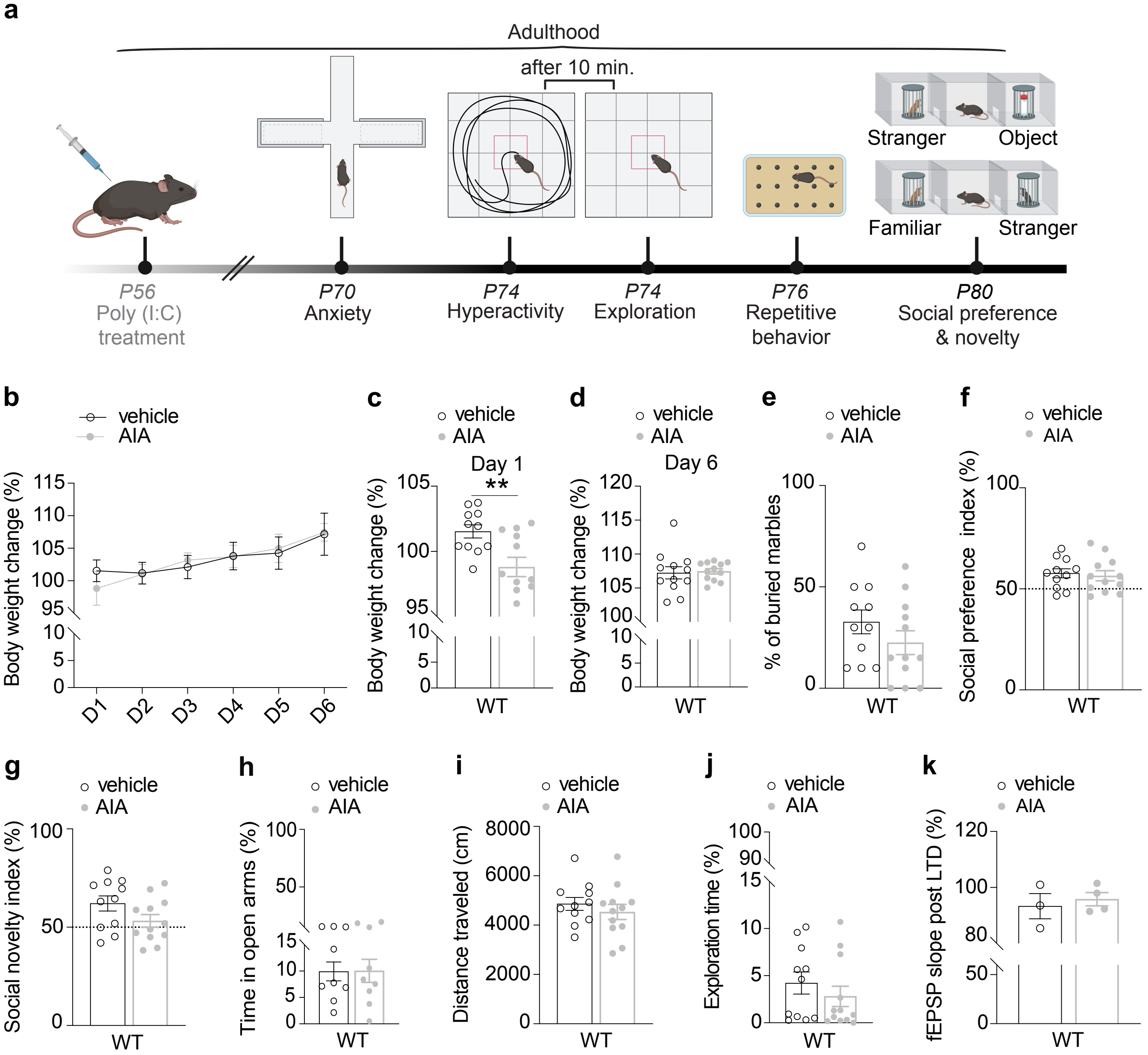

Supplementary Figure 5

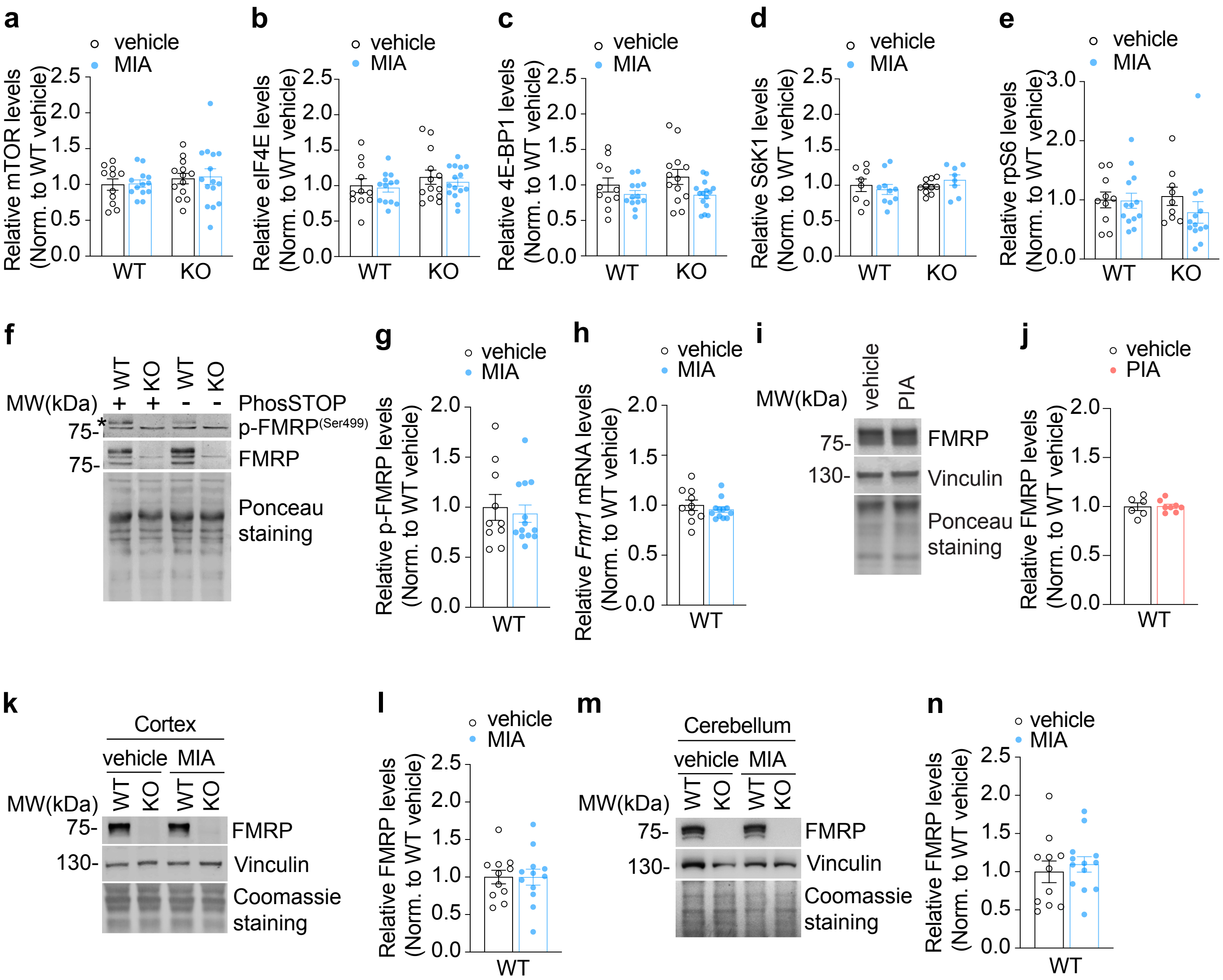

Supplementary Figure 6

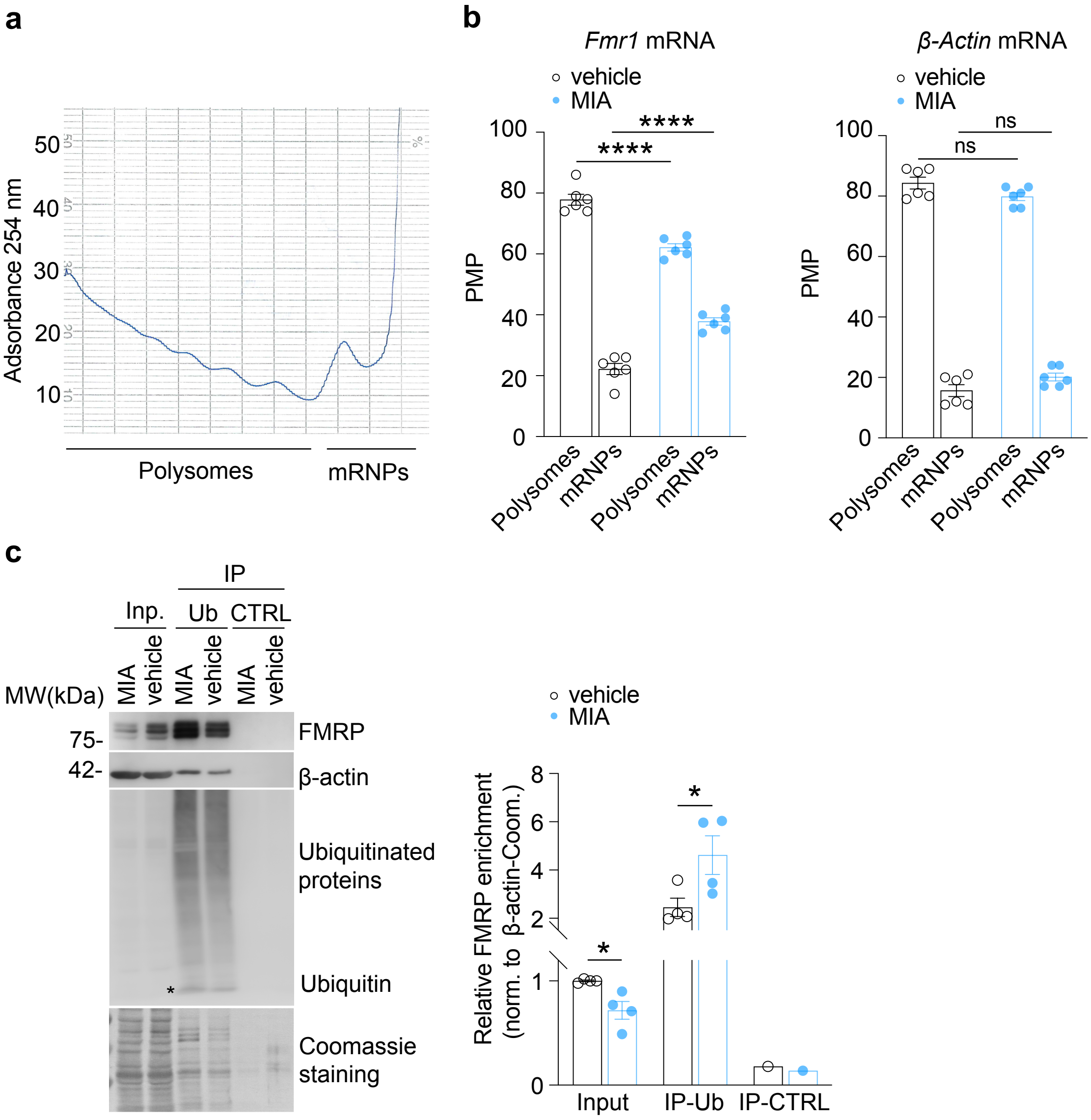

Supplement: Supplementary file 1 — Hilal Rosina et al suppl material [file 41380_2024_2805_MOESM1_ESM.pdf]
